# Next-generation full-thickness human skin models produced using 3D electrospun scaffolds and animal-component-free culture media

**DOI:** 10.3389/ftox.2026.1639389

**Published:** 2026-05-13

**Authors:** Patrick J. Hayden, Jayashree Chakravarty, Rayan K. Hodroj, Sar R. Lindner, Lisa M. Fitzgerald, Jacob T. Le Do, Hsin Tzu Keng, Vivek Patel, Holger P. Behrsing, Matthew D. Phaneuf

**Affiliations:** 1 BioSurfaces, Inc., Ashland, MA, United States; 2 Electron Microscopy Facility, Harvard University, Boston, MA, United States; 3 Institute for In Vitro Sciences, Inc., Gaithersburg, MD, United States

**Keywords:** animal-free culture medium, electrospun scaffold, fetal bovine serum replacement, full-thickness skin model, *in vitro* skin model

## Abstract

**Introduction:**

In vitro full-thickness human skin (FT-Skin) models are important tools for testing of cosmetics and chemicals, screening of new pharmaceuticals, and human disease modeling research. However, these skin models commonly utilize animal-derived collagen as a main structural element of the stromal matrix. Animal-derived collagen constructs suffer from stability and contraction issues, resulting in short lifespan and poor reproducibility. Additionally, culture media utilized to produce these models commonly contain undesirable animal-derived components including fetal bovine serum (FBS) and bovine pituitary extract (BPE).

**Methods:**

To address these shortcomings, FT-Skin models were developed without animal-derived collagen by using electrospun scaffolds as a structural component of the stromal constructs, together with FBS- and BPE-free culture media formulations.

**Results:**

These novel culture protocols and media formulations produced well-developed FT-Skin models with improved lifespan and barrier properties. Protocol transferability, and intra- and inter-lot reproducibility of the FT-Skin models were demonstrated by testing in 2 independent laboratories.

**Discussion:**

These next-generation FT-Skin models offer opportunities for completely animal-product-free testing of cosmetics and chemicals, screening of new pharmaceuticals and more human-relevant modeling of skin diseases. The electrospun scaffolds and basic processes for development of animal-free subepithelial stromal constructs are also anticipated to be adaptable to the development of additional epithelial tissue models such as ocular, airway and intestine.

## 1 Introduction

*In vitro* models that reproduce key 3-dimensional (3D) structural and organotypic functions of *in vivo* human skin are increasingly important for a variety of international regulatory testing applications, as well as basic research applications in skin biology and pharmaceutical development (Hayden et al., 2016; Kandarova and Hayden, 2021; Zhao et al., 2023). Two broad categories of *in vitro* skin models include “partial-thickness” models that are comprised of only an epithelial component, and “full-thickness” models that are comprised of both epithelial and sub-epithelial stromal components. Dermal-epidermal interactions play a key role in regulation of epidermal proliferation, differentiation, wound healing and barrier function, and are involved in the pathogenesis of aging/photoaging, immune responses and numerous skin diseases, (Smola et al., 1993; El Ghalbzouri et al., 2005; Werner et al., 2007; Varkey et al., 2014). Full-thickness skin (FT-Skin) models therefore have the potential to provide more comprehensive and *in vivo*-relevant experimental data compared to partial-thickness models.

*In vitro* FT-Skin models commonly utilize collagen derived from rat, bovine or porcine sources as a main structural element of a 3-dimensional (3D) dermal matrix. Alternatively, human fibroblasts cultured in growth medium supplemented with 2-phospho-L-ascorbic acid may also be used to produce self-assembled extracellular matrix that functions as the dermal component for FT-Skin model production (Hata and Senoo, 1989; Michel et al., 1999). However, FT-Skin constructs produced by either of these approaches have suffered from stability and contraction issues, resulting in short lifespan and poor reproducibility of the models (Stark et al., 2006; Sanchez and Morgan, 2021; Tan et al., 2022; Tham et al., 2025). Additionally, culture media utilized to produce FT-Skin models commonly contain animal-derived components including fetal bovine serum (FBS) and bovine pituitary extract (BPE). The use of animal collagen and other animal-derived components to produce these models is problematic due to scientific issues including questionable human relevance, potential introduction of pathogens or infectious agents and batch-to-batch variability, as well as the ethical issue of pain and suffering inflicted upon the animals during harvest or isolation of the materials (Rauch et al., 2011; van der Valk et al., 2018; Weber and Wagner, 2021; Weber et al., 2025).

**FIGURE 1:**
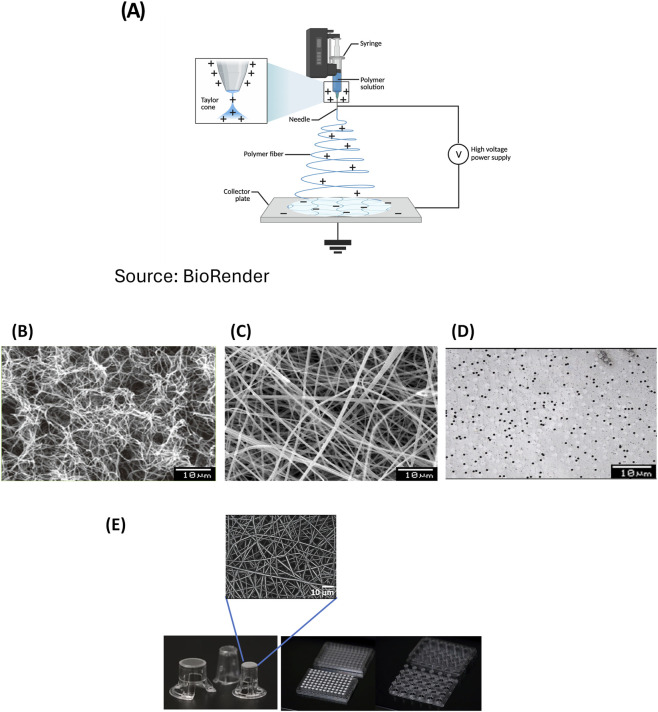
Electrospun Scaffolds Compared to Natural *In Vivo* Extracellular Matrix and Common Microporous Membranes. **(A)** Schematic depiction of the solution electrospinning process. Polymer solution is pumped though a needle and subjected to a high-voltage electric field. The field draws the solution into a fine jet, which elongates and thins as the solvent evaporates, leaving behind solid polymer nanofibers. These nanofibers are collected on a grounded surface, forming a nonwoven mat with nanoscale diameters and tunable properties (from Wagay, D. (2023); Wagay, D. (2026) BioRender). Scanning electron micrographs of **(B)** *in vivo* extracellular matrix (from Patrawalla et al., 2023); **(C)** 3D Bio-Spun® PET scaffold. Ave. fiber diameter = 2 +/- 1 μm. Ave. pore size = 3+/- 2 μm (BioSurfaces); and **(D)** 2D film-based PET microporous membrane (Sterlitech). **(E)** Electrospun scaffolds are bonded to various sizes and formats of inserts (shown in the upside-down orientation to highlight the electrospun scaffold component, BioSurfaces). Scale bars = 10 μm.

The limitations of dermal constructs as noted above have motivated efforts to develop alternative structural scaffolds for FT-Skin model production. Most efforts have focused on development of various types of natural and synthetic hydrogel scaffolds (Tan et al., 2022) and strategies for improving their mechanical properties, often combined with novel bioprinting techniques (Shi et al., 2026). A related alternative approach involves electrospinning, a process that has shown utility in producing scaffolds for a variety of biomedical and tissue engineering applications (Jun et al., 2018; Tsareva et al., 2025) (Figure 1A). The nanoscale fibrous architecture of electrospun scaffolds mimics native extracellular matrix (ECM), providing a functional 3D microenvironment that supports cellular adhesion, proliferation, and tissue regeneration (Figure 1). Among the most common materials used for producing electrospun scaffolds are synthetic polymers such as polyethylene terephthalate (PET) (Imaichi-kobayashi et al., 2023), polylactic acid (PLA), polyglycolic acid (PGA), poly (lactic-coglycolic acid) (PLGA), and poly (ε-caprolactone) (PCL), as well as natural biopolymers such as collagen, silk fibroin, and chitosan, (Jun et al., 2018). Electrospun polyamide scaffolds have also been utilized in efforts to produce FT-Skin models (Weigel et al., 2022).

In addition to the ethical concerns associated with the use of animal-derived products, development of cell culture media that are free of animal-derived components (xeno-free) has been driven by the need for safer and more consistent products for biomedical research and clinical applications (Rajala et al., 2010; Chase et al., 2012; Panella et al., 2021; Hua et al., 2022; Weber et al., 2024; Oredsson et al., 2025). By eliminating animal-derived components, xeno-free media reduce the risk of pathogen transmission and meet the stringent regulatory standards for cell-based therapies. Additionally, xeno-free conditions often improve cellular behavior and are better suited for advanced technologies such as gene editing and personalized medicine. A variety of commercial xeno-free cell culture media products are now available in response to these needs. Of specific relevance to *in vitro* skin models, LifeLine Cell Technology (Frederick, MD) offers Xeno-free fibroblast medium containing human serum in place of FBS, and CELLnTEC Advanced Cell Systems (Bern, CN) offers proprietary defined keratinocyte expansion and differentiation media. Baltazar et al. (2022) has reported progress in development of a bioprinted implantable FT-Skin graft produced entirely of xeno-free components.

To address both material and ethical issues associated with existing *in vitro* FT-Skin model production approaches, the current work undertook development of FT-Skin models using commercially available synthetic electrospun scaffolds (Bio-Spun®) as a structural dermal element (BioSurfaces, Ashland, MA). The scaffolds are produced by a solution electrospinning process using synthetic medical grade PET and PLLA/PDLGA polymers that are FDA-approved for use in medical device applications. When populated with human dermal fibroblasts, a fully human extracellular matrix was established within the electrospun scaffolds, thereby eliminating the need for animal-derived collagen as a component. Because the 3D electrospun scaffold is an integral part of the dermal component, issues with detachment from the insert, contraction and subsequent matrix degradation were eliminated, thereby providing improved stability and lifespan to the models.

FBS- and BPE-free culture media formulations were also developed, providing new opportunities in the direction of completely animal-free production of *in vitro* human FT-Skin models. Together, the use of electrospun scaffolds and use of FBS- and BPE-free medium formulations eliminates ethical concerns of animal product use, and provides improved human relevance as well as improved lifespan and stratum corneum barrier. Furthermore, the open-source protocols and detailed media formulations reported here offer opportunities for decreased reliance on proprietary commercial skin models, and were demonstrated to be highly transferable and reproducible within and between laboratories.

## 2 Materials and methods

### 2.1 Chemicals

2-Phospho-L-ascorbic acid trisodium salt (Cat. #49752), arachidonic acid (Cat. #181198), isoproterenol HCl (Cat. #I5627), L-carnitine (Cat. #C0158), linoleic acid (Cat. #L1012), L-serine (Cat. #84959), oleic acid (Cat. #O1383), palmitic acid (Cat. #P5585), (+/−)-α-tocopherol (Cat. #T3251), 3-(4,5-Dimethyl-2-thiazolyl)-2,5-diphenyl-2H-tetrazolium bromide (MTT, Cat. #M5655) and dimethylsulfoxide (DMSO, Cat. #D2650-5X5ML) were from Sigma-Aldrich (St. Louis, MO). GM 6001 (Cat. #HY-15768) was from MedChemExpress (Monmouth Junction, NJ). 2-(2-methoxy-4-nitrophenyl)-3-(4-nitrophenyl)-5-(2,4-disulfophenyl)-2H-tetrazolium, monosodium salt (WST-8, CCK-8 Kit, Cat. #CK04) was from Dojindo Molecular Technologies, Inc. (Gaithersburg, MD). Triton X-100 was from Boston Bioproducts (Milford, MA). All of the above chemicals were derived from non-animal sources.

### 2.2 Base culture media, reagents and supplements

Human Keratinocyte Growth Supplement (HKGS, Cat. #S0015), Supplement S7 (Cat. #S0175), Medium 154 (Cat. #M154500), EpiLife Medium (Cat. #MEPI500CA), DMEM, high glucose (Cat. #11965-084), Antibiotic-Antimycotic solution (Cat. #R01510 or #15240062, both are animal-origin free). TrypLE™ Express (Cat #12605010), Trypan Blue Solution, 0.4% (Cat #15250061) and Synth-a-Freeze cryopreservation medium (Cat #A1254201) were obtained from ThermoFisher Scientific (Waltham, MA). Fibroblast Growth Medium-2 bullet kits (FGM-2, Cat #CC-3132) and KGM® Gold keratinocyte growth medium bullet kits (Cat # 00192060) were obtained from Lonza (Walkersville, MD). Ca^2+^/Mg^2+^-free Dulbecco’s phosphate buffered saline (DPBS, Cat # SH30028.03) was obtained from Cytiva (Marlborough, MA). Bovine serum albumin (BSA) fraction V fatty acid-free (Cat. #A-421-100) was from GOLDBIO (St. Louis, MO). Transforming growth factor beta-1 (TGF-β1, Cat. #100-21) and connective tissue growth factor (CTGF, Cat. #120-19) were from PeproTech (ThermoFisher). Xeno-free human platelet lysate (HPL) was from StemCell Technologies (Vancouver, BC, Cat. #200-0360) or Sexton Biotechnologies (Indianapolis, IN, Cat #PL-CP-NH-100). Human serum (Cat. #H6914) was from Sigma-Aldrich.

### 2.3 FT-skin model medium formulations

Details of media development/optimization experiments can be found in the Supplementary Material. Finalized medium formulations are given below.

#### 2.3.1 Dermal development medium (DDM)

##### 2.3.1.1 DDM with FBS

DDM consisted of Fibroblast Basal Medium (FBM, Lonza) supplemented with insulin, human fibroblast growth factor-B (hFGF-B), gentamicin and amphotericin (FGM-2 Bullet Supplements, Lonza) in amounts provided by the supplier. The standard FGM-2 bullet supplements as provided by Lonza also include fetal bovine serum (FBS). FBS-containing DDM was utilized in initial experiments but was subsequently replaced with HPL as described below.

##### 2.3.1.2 Replacement of FBS in FGM-2 and DDM

Beginning with cryopreserved passage 3 (P3) NHDF (previously expanded in FGM-2 with FBS), the cells were serially passaged to P5 in FGM-2 containing various concentrations of HPL in place of FBS. Standard FGM-2 containing 2.0% FBS was used as a comparative control condition. HPL concentrations of 2.5, 1.0, 0.5 and 0.25% were evaluated. Cells were seeded at an initial density of 6,667/cm^2^. NHDF were counted at each passage before re-seeding at the same initial density. All cells were harvested and cryopreserved at P5. Experiments described in Sections 3.4–3.6 utilized DDM formulated with HPL in place of FBS.

#### 2.3.2 Epidermal submerged medium (ESM)

ESM was prepared from Medium 154 or EpiLife Medium supplemented with either HKGS or Supplement S7 (1/100 dilution in base medium) as indicated for specific experiments described in the Results section. The medium was further supplemented with 2-phospho-L-ascorbic acid (50 μg/mL). HKGS consists of bovine pituitary extract (BPE, 0.2%), recombinant human insulin-like growth factor-I (rhIGF-I, 0.01 μg/mL), hydrocortisone (HC, 0.18 μg/mL), bovine transferrin (5 μg/mL), human epidermal growth factor (EGF, 0.2 ng/mL), gentamicin and amphotericin. The Supplement S7 formulation is proprietary (ThermoFisher). ESM prepared with HKGS was utilized in initial experiments, but was replaced with animal-free supplement S7 for experiments described in Section 3.6. Because Supplement S7 does not contain gentamicin and amphotericin, Antibiotic-Antimycotic solution (ThermoFisher) may be optionally added.

#### 2.3.3 Epidermal differentiation medium (EDM)

EDM base medium consisted of a 50:50 mixture of high-glucose DMEM and either Medium 154 or EpiLife Medium, as indicated for specific experiments described in the Results section. Base media were supplemented with HKGS (1/100 dilution) for initial experiments, but was replaced with animal-free supplement S7 (1/100 dilution) for experiments described in Section 3.6. Both HKGS- and Supplement S7-containing EDM were further supplemented with fatty acid-free BSA (FAF-BSA, 0.1%), L-serine (10 mM), L-carnitine (10 μM), arachidonic acid (7 μM), linoleic acid (15 μM), oleic acid (25 μM), palmitic acid (25 μM) and (+/−)-α-tocopherol (22 μM) as described by Boyce and Williams (1993), as well as 2-phospho-L-ascorbic acid (50 μg/mL), isoproterenol (1.0 μM) and GM6001 (2 μM). These EDM formulations maintained stability when stored at 4 °C for at least 6 weeks. All chemicals and supplements with the exception of the FAF-BSA were of animal-free origin. FAF-BSA was utilized instead of FAF-human serum albumin (HSA) due to cost constraints of performing the current work.

### 2.4 Cells

#### 2.4.1 Cell sources and culture methods

Normal human dermal fibroblasts (NHDF-Neo, Cat. #CC-2509 or NHDF-Ad, Cat. #CC-2511) were obtained from Lonza. Human epidermal keratinocytes were from ThermoFisher Scientific (HEKn, Cat. #C0015C) or Lonza (NHEK-Neo, Cat. #00192907 or NHEK-Ad, Cat. #00192627). Complete donor information is provided in Supplementary Table S1. Cryopreserved cells were obtained at passage 1 (P1), and expanded prior to use for skin model production following the suppliers’ protocols. Generally, vials of cryopreserved cells were rapidly thawed in a 37 °C water bath. Thawed cell suspensions were diluted to 16,667 cells/mL in growth medium and plated at ∼3,333 cells/cm^2^ into tissue culture treated flasks. The following day, the medium was removed and the cells were fed with fresh growth medium. Cells were fed again every other day until they reached 70%–80% confluence, at which time they were harvested for further passage or cryopreservation.

NHDF were expanded in FGM-2 containing either FBS or HPL as indicated above in Section 2.3.1.2 and Results Section 3.3. HEKn/NHEK donors were expanded in EpiLife supplemented with HKGS (for HEKn) or KGM Gold supplemented with KGM Bullet Supplements including BPE (for NHEK).

#### 2.4.2 Harvest of cells for passage or cryopreservation

For NHDF, growth medium was removed and cultures were rinsed with DPBS. Cells were then detached with TrypLE™ Express (5 mL/75 cm^2^ **)**. For harvest of NHEK/HEKn, growth medium was removed and cultures were rinsed with DPBS followed by an additional 5 min soak in DPBS (15 mL/75 cm^2^) at 37 °C. After removal of the DPBS soak, cells were detached with TrypLE™ Express (5 mL/75 cm^2^ **)**. Both NHEK/HEKn and NHDF were effectively detached by TrypLE™ Express within about 5 min at 37 °C. Once detached, cells were diluted in an equal volume of DPBS, collected and centrifuged at 200 *g*. The cell pellets were resuspended in growth medium for passaging. For cryopreservation, cell pellets were resuspended in either Synth-a-Freeze™ (Gibco) or medium containing 10% DMSO and 10% human serum as noted below. In both cases, cells were counted using a hemocytometer, and viability of harvested cells was determined by the Trypan Blue exclusion method following the supplier’s protocol (ThermoFisher).

#### 2.4.3 Cryopreservation methods

Synth-a-Freeze™ cryopreservation medium was utilized for cryopreservation of cells at ≤ 3 million cells/mL. Cell suspensions were dispensed into 1.0 mL cryovials, placed in a Mr. Frosty freezing chamber (ThermoFisher) containing isopropanol, and transferred to −80 °C overnight, followed by transfer to liquid nitrogen vapor phase for long-term storage. In some cases, cells at final passage of P6 for NHDF and P4 for HEKn or NHEK were cryopreserved at high cell concentrations (in the range of 3.0–7.0 million cells/mL) so they could be seeded directly onto the scaffolds upon thawing without additional expansion. Synth-a-Freeze^TM^ non-protein cryopreservation medium is not recommended for use at cell concentrations greater than 3 million/mL, and was found to provide sub-optimal recovery and cell viability at higher cell concentrations. Therefore, cryopreservation medium containing 10% DMSO and 10% human serum was utilized in place of Synth-a-Freeze^TM^, using the same harvest and freezing protocols as described above.

For seeding directly onto electrospun scaffold inserts without final expansion, cryopreserved cells were rapidly thawed at 37 °C and diluted 10-fold into growth medium, followed by centrifugation at 200 × g for 10 min to remove the cryopreservation medium. Cell pellets were subsequently resuspended in growth medium at a concentration adequate for directly seeding onto Bio-Spun® inserts (generally 1-2 million cells/mL). Viability of cells following recovery from cryopreservation was determined by the Trypan Blue exclusion method following the supplier’s protocol (ThermoFisher). Viable cell recoveries of NHDF and HEKn/NHEK above 80% were routinely achieved by this process. It is recommended to add slightly more cells per vial than necessary to avoid running short when seeding onto inserts. NHDF and HEKn/NHEK cryopreserved by this process were used in experiments described below in Sections 3.4–3.6.

### 2.5 Electrospun scaffold inserts and lifter plates

24-well high throughput screening (HTS) and 24-well individual insert formats containing Bio-Spun® PET scaffolds (150 μm thickness, Cat. #WP24-200 and Cat. #11C24-200, respectively), and 24-well HTS and 24-well individual insert formats containing Bio-Spun® PLLA/PDLGA bilayer scaffolds (100 μm thickness, Cat. #WP24-502 and Cat. #ILC24-502, respectively) were from BioSurfaces, Inc. (Ashland, MA). These inserts incorporate electrospun nanofiber scaffolds in place of the film-based track-etched pore membranes found in traditional insert products.

The Bio-Spun® PET scaffolds are fabricated from biocompatible polymers that are FDA-approved for use in clinical medical devices. The PET scaffold is non-degradable and has an average fiber diameter of 2 +/− 1 μm and effective pore size of 3 +/− 2 μm. The Bio-Spun® bilayer PDLGA/PLLA scaffold is biodegradable. The lower layer facing the basolateral side of the insert is composed of PLLA (fiber diameter = 0.6 ± 0.2 μm), while the upper layer facing the apical surface of the insert is composed of PDLGA (fiber diameter = 2 ± 1 μm). The effective pore size of the PDLGA/PLLA scaffold is 3 +/− 2 μm and 2 +/− 1 μm for the PDLGA and PLLA layers, respectively. The PDLGA layer will show significant degradation within 4–5 weeks of culture, while the PLLA layer will degrade slowly over a period of months. The scaffolds may be plasma treated by the end user to increase hydrophilicity and promote cell adhesion. However, plasma treatment may induce partial degradation of the polymers and is not necessary when the scaffolds are activated as described below in Section 2.6.

**FIGURE 2:**
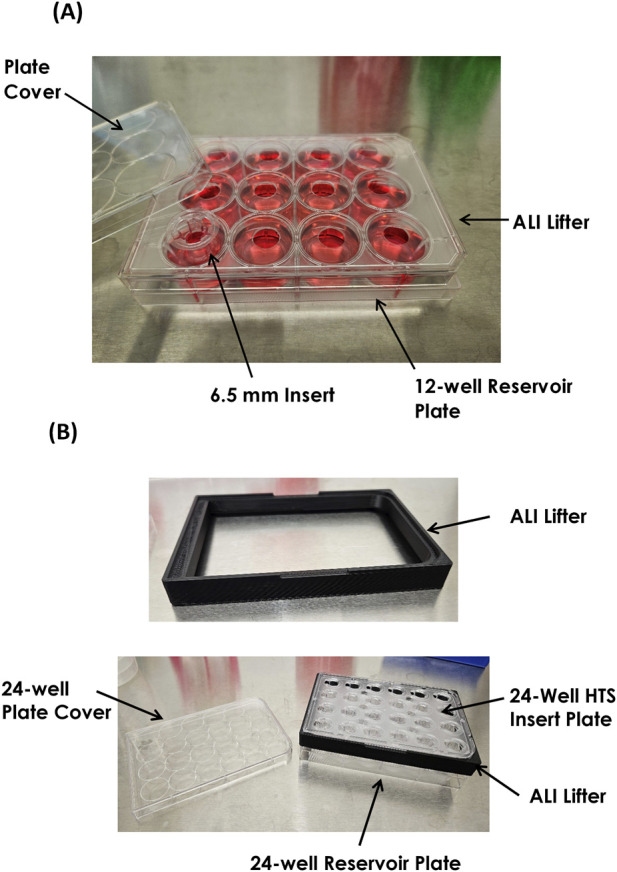
Air-Liquid Interface (ALI) Lifters. ALI lifters enable additional culture medium beneath the scaffolds during the ALI culture period. **(A)** 6.5 mm diameter scaffold inserts are transferred from the standard 24-well plate to a 12-well plate in combination with the 6.5 mm insert lifter (BioSurfaces). The lifter fits between the plate and the plate cover. This allows feeding with 5.0 mL/well of medium. **(B)** The 24-well HTS ALI lifter fits between the 24-well HTS plate and plate cover (BioSurfaces). This allows feeding with 3.0 mL/well of medium.

Specialized air-liquid interface (ALI) insert lifters were utilized during the ALI culture phase to allow additional culture medium to be dispensed beneath the scaffolds and provide sufficient media volume for extended periods between required feedings (Figure 2). Air-liquid interface (ALI) insert lifters for 24-well HTS and 24-well individual insert formats (Cat. #ALIE-HTS24 and Cat. #ALI-LC24, respectively) were also from BioSurfaces. Inserts and lifter plates were sterilized with UVC.

### 2.6 Scaffold insert preparation

#### 2.6.1 Scaffold activation – PET scaffolds

The electrospun scaffolds require pre-treatment to wet the scaffold fibers and promote cell attachment. For the PET scaffolds, this is accomplished by first submersing the scaffolds in a solution of 50% ethanol in water for 1 h at 37 °C (USP grade ethanol and tissue culture grade H_2_O). For 6.5 mm diameter inserts (24-well size), 1.5 mL of ethanol solution is added to the scaffold plate wells so that the solution fills the inserts by passing though the scaffold from below. The 50% ethanol displaces air from between the scaffold fibers and facilitates wetting of the PET fibers. After the 1-h exposure, the 50% ethanol solution is replaced with 100% H_2_O and the scaffolds are further conditioned for at least 3 h at 37 °C. For convenience, the inserts may be wetted in advance and left soaking in the 100% H_2_O overnight or over the weekend prior to use. Following the wetting period, the H_2_O is removed and the scaffolds are further conditioned in DDM for at least 1 h at 37 °C. Following the conditioning period, the DDM is removed and replaced with fresh DDM immediately prior to seeding NHDF. The DDM may contain either FBS or HPL as described for specific experiments in sections below.

#### 2.6.2 Scaffold activation – PDLGA/PLLA scaffolds

Ethanol-containing wetting solutions are utilized only for PET scaffolds and should not be applied to PDLGA/PLLA scaffolds, as the ethanol will initiate degradation of the polymers. The PDLGA/PLLA scaffolds are easily wettable, and are conditioned directly in DDM for at least 1 h at 37 °C. Following the conditioning period, the DDM is removed and replaced with fresh DDM immediately prior to seeding NHDF.

### 2.7 FT-skin model production protocols

#### 2.7.1 General FT-skin model production protocol

**FIGURE 3:**
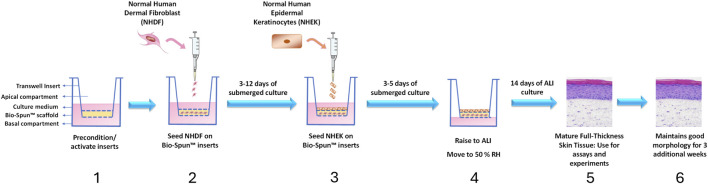
Schematic workflow for General FT-Skin model production. 1: NHDF are first seeded onto the apical surface of the electrospun scaffold insert and cultured under submerged conditions. The NHDF migrate into the scaffold and secrete human ECM. A robust dermal component is produced within 3–12 days of submerged culture, depending on the initial NHDF seeding density. 2: NHEK are then seeded onto the dermal component and cultured under submerged conditions for 3–5 days in Epidermal Submerged Medium (ESM) to initiate formation of the epidermal component. 3: The cultures are then raised to the air-liquid interface (ALI) and cultured for an additional 14 days in Epidermal Differentiation Medium (EDM). 4: The fully mature FT-Skin tissue is ready to use by day 14. 5: The FT-Skin tissue may be cultured for at least three additional weeks with maintenance of good epidermal thickness and morphology.

A general protocol based on common methods for *in vitro* FT-Skin model production was utilized during the initial phases of the current work to evaluate variables such as cell seeding densities, culturing periods and media conditions (Figure 3). The DDM utilized for these experiments contained either FBS or HPL, and the ESM and EDM utilized for these experiments contained either HKGS or Supplement S7 as described for specific experiments in the Results sections below.

A critical aspect for success of the FT-Skin model is development of a robust dermal component. Following scaffold activation, NDHF are seeded onto the apical surface of the scaffolds and cultured under submerged conditions in DDM. During this culture period, the fibroblasts migrate into the scaffold, where they attach, proliferate and secrete natural dermal extracellular matrix (ECM) materials. The presence of deposited ECM may be confirmed by staining with Van Gieson’s stain (Section 2.8; Supplementary Figure S4). Deposition of ECM and development of a robust dermal component is dependent on the number of fibroblasts seeded and the duration of dermal development time. The optimum seeding density and culture time will be donor dependent, especially for NHDF that are derived from adult donors, for which proliferation rates and matrix secretion capabilities may be diminished by age (Costello et al., 2022).

In practical terms, the robustness of the dermal component is defined by its ability to prevent subsequently applied keratinocytes from migrating into the scaffold. As a visual cue during the dermal development process, when culture medium is aspirated from the wells beneath the scaffold inserts, the dermal component formed within the electrospun scaffold should prevent medium still contained within the apical compartment of the insert from leaking through into the well below. If the inserts do not retain the culture medium, the matrix deposition is likely inadequate to support good epidermal development. Further optimization of dermal development for the specific donor lot (i.e., addition of more NHDF or longer culture time) should be performed before proceeding. Otherwise, the HEKn will migrate into the scaffold rather than forming a well-organized epithelium above the dermal scaffold (Supplementary Figure S5).

#### 2.7.2 3-Week FT-skin model production protocol

Though an iterative evaluation process, the time required to generate a dermal component capable of supporting further development of the FT-Skin tissue model was reduced to only 3 days. This was achieved by increasing the NHDF seeding density to 175,000/insert (530,303 cells/cm^2^ for a 6.5 mm diameter 24-well size insert). NHDF (donor 19TL149597, final passage P6) and HEKn (donor 2491219, final passage P4) were cryopreserved at high cell concentrations and seeded directly after thawing as described in Section 2.4.3 above. The NHDF were previously expanded from P3 to P6 using FGM-2 containing HPL in place of FBS (Section 2.3.1.2). DDM also used 2.0% HPL in place of FBS. Both NHDF and HEKn were cryopreserved in medium containing 10% human serum in place of FBS. Large freeze-lots of the high concentration NHDF and HEKn were prepared so that each freeze-lot could be pre-qualified and used to produce multiple FT-Skin tissue lots (ideally, 10 or more FT-Skin tissue lots can be produced from each pre-qualified NHDF and HEKn freeze-lot). Viable cell recoveries were determined for the pre-qualified cryopreserved NHDF and HEKn cell lots. Therefore, in order to minimize cell handling and time during the production process, counting of the viable cells post-thaw was not routinely performed for each production lot of FT-Skin tissues. The cell numbers used in the protocol assumed and accommodated a viable cell loss of up to 20% from the thawing/recovery process.

**FIGURE 4:**
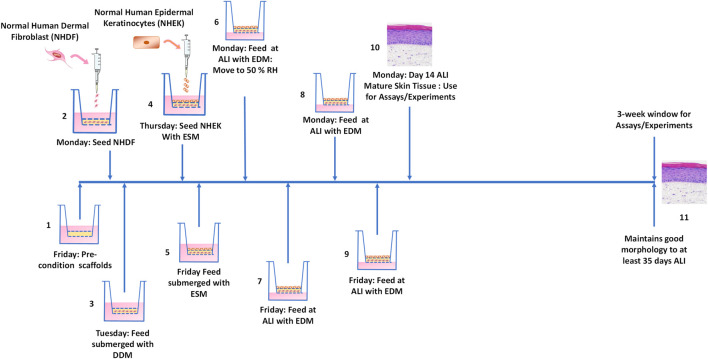
Schematic workflow for 3-week FT-Skin model production. The FT-Skin production protocol minimizes the production time and feedings, and eliminates weekend feedings. NHDF and HEKn/NHEK are seeded directly after thawing without final expansion. Mature FT-Skin tissues are ready on a Monday schedule to maximize experimental assays time during the week. 1: Scaffolds are activated by pre-wetting on Friday. 2: NHDF are seeded onto the apical surface of the electrospun scaffold on Monday under submerged culture conditions. 3: Dermal components are fed under submerged conditions on Tuesday with DDM. 4: NHEK are seeded onto the dermal component and cultured under submerged conditions in Epidermal Submerged Medium (ESM) to initiate formation of the epidermal component on Thursday. 5: FT-Skin tissues are fed under submerged conditions with ESM on Friday. 6: The developing FT-Skin tissues are raised to the air-liquid interface (ALI) in Epidermal Differentiation Medium (EDM) on Monday. Cultures are also transferred to 50% relative humidity (RH) incubator. 7, 8, 9: The developing FT-Skin tissues are fed at the ALI on the following Friday, Monday and Friday for 24-well individual insert formats. The 24-well HTS insert formats are fed on Wednesdays as well. 10: The fully mature FT-Skin tissues are ready to use the following Monday at day 14 of ALI culture. 11: The FT-Skin tissues may be cultured for at least three additional weeks with maintenance of good epidermal thickness and morphology.

Seeding and feeding schedules were chosen to minimize handling, avoid weekend feedings, and provide mature FT-Skin tissues on a Monday schedule for optimum utility in downstream assays and experimental applications. Because the ALI lifter and culture plates used for the 24-well individual inserts can accommodate 5.0 mL/well, this format allows for feeding on Mondays and Fridays only. The 24-well HTS format can accommodate only 3.0 mL/well for ALI culture, so is fed on Mondays, Wednesdays and Fridays. The entire production process for both formats is accomplished in 3 weeks, excluding the pre-wetting step which can be conveniently initiated on Friday prior to the start of the culture period. The complete production schedule involves the following steps as depicted in Figure 4 and described in detail below.

##### 2.7.2.1 Seeding of NHDF and development of the dermal component

On the Monday following scaffold activation, inserts were equilibrated for 1 h at 37 °C by addition of 1.5 mL of DDM (formulated with HPL in place of FBS) to the well beneath each insert. Immediately prior to seeding the NHDF, the DDM was aspirated from both the inserts and the wells, and replaced with a fresh 1.0 mL of DDM beneath each insert. One vial of high concentration cryopreserved cells (4.5 million cells per vial in 1.0 mL) was thawed, diluted into 9.0 mL of DDM and centrifuged at 200 × g for 10 min. The cell pellet was re-suspended in DDM to give a concentration of 1 × 10^6^ cells/mL. NHDF suspensions were then applied to the apical surfaces of the inserts in a volume of 175 μL. Following this procedure, 4.2 million NHDF are required to seed 24 inserts. Immediately following seeding of the NHDF, and additional 0.5 mL/well of DDM was added to the wells beneath each insert. The day after NHDF seeding, the DDM was aspirated from both the top and bottom of the inserts, and the tissues were fed submerged with 2.5 mL/well of DDM.

On the Thursday following NHDF seeding, the DDM was aspirated from beneath each insert scaffold. The dermal component formed within the electrospun scaffold was developed enough to prevent the medium still contained within the apical compartment of the insert from leaking into the well beneath the insert. The remaining DDM contained within the apical compartment was then aspirated using a Pasteur pipet. The presence of deposited ECM was further confirmed by staining with Van Gieson’s stain (Supplementary Figure S4).

##### 2.7.2.2 Seeding of HEKn and submerged epithelial culture

Following removal of DDM from the wells beneath each insert and the apical insert compartment, 1.0 mL/well of ESM (Formulated with either HKGS of Supplement S7 as described for specific experiments) was dispensed into the wells beneath each insert. One vial of high concentration cryopreserved HEKn (6.0 million cells per vial in 1.0 mL) was thawed, diluted into 9.0 mL of ESM and centrifuged at 200 × g for 10 min. The cell pellet was re-suspended in ESM to give a concentration of 2 × 10^6^ cells/mL. HEKn suspensions were then applied to the apical surfaces of the dermal component in a volume of 125 μL. Immediately following seeding the HEKn, an additional 0.5 mL/well of ESM was added to the wells beneath each insert. The day after HEKn seeding (Friday), the ESM was aspirated from both the top and bottom of the inserts, and the tissues were fed submerged with 2.5 mL/well of ESM.

##### 2.7.2.3 Air-liquid interface (ALI) culture

On Monday following seeding of HEKn, the cultures were raised to the ALI. This involves raising the scaffolds with ALI lifters to allow larger amounts of medium to be dispensed into the reservoir wells below each insert (Figure 2), and prolong the required interval between medium changes (Figure 4). EDM was formulated with either HKGS or Supplement S7 as described for specific experiments. Additionally, concurrent with the initiation of ALI culture, the tissues are moved to a 37 °C, 5% CO_2_ incubator with relative humidity (RH) lowered to 50% in order to improve the barrier lipid synthesis and epidermal barrier as previously reported by Sun et al. (2015).

For the 24-well (6.5 mm diameter) individual insert format, 5.0 mL of EDM per well were dispensed into each well of the 12-well plate. The ALI lifter (Figure 2A) was applied to the plate and the inserts were transferred from the 24-well plate into the 12-well ALI plate using sterile forceps. In the case of the 24-well-HTS format, 3.0 mL of EDM were dispensed into each well of a fresh 24-well reservoir plate before insertion of the 24-well-HTS lifter (Figure 2B), and transfer of the scaffolds to the fresh plate. No medium was added to the apical surface of the tissues during the ALI culture period. ALI culture continued with feeding every Monday and Friday for the 24-well individual format, or every Monday, Wednesday and Friday for the 24-well-HTS format, according to the schedule outlined in Figure 4. FT-Skin tissues were considered fully mature after 14 days at the ALI (i.e. the 2nd Monday following initial ALI), based on histological assessment and stratum corneum development. Following maturation (day 14 ALI), culture is continued for the desired duration of the end-use experiment, up to at least 3 additional weeks.

### 2.8 Histology processing and staining

FT-Skin model samples were fixed in 10% neutral buffered formalin for 2 h at RT. Fixed tissues were rinsed in DPBS and dehydrated by a series of washes in 95% and 100% ethanol (2 washes each for 15 min at 58 °C–60 °C), then cleared with xylene (1 wash for 15 min at 58 °C–60 °C) prior to immersion in molten paraffin (1 time for 15 min, then 1 time overnight at 58 °C–60 °C. Paraffin-infused tissues were then embedded in molten paraffin using a mold and cooled to obtain solid paraffin-embedded tissue blocks. Five μm cross-sections were cut from the blocks and mounted onto polylysine-coated glass slides. Slides were heated for 2 h at 58 °C–60 °C prior to deparaffinization and rehydration by a series of rinses in xylene (3 times for 1 min), 100% ethanol (3 times for 1 min), 95% ethanol (3 times for 1 min), 80% ethanol (1 time for 1 min) and water (1 time for 1 min), and stained with hematoxylin and eosin (H&E) or Van Gieson’s stain following the supplier’s protocols (Newcomer Supply, Waunakee, WI). Images and quantitative measurements were obtained on an Olympus IX51microscope equipped with CellSens imaging and analysis software.

### 2.9 Immunofluorescence microscopy (IF)

FT-Skin model samples were fixed in 10% neutral buffered formalin for 2 h at RT prior to paraffin embedding as described above in Section 2.8 for histology processing. Five μm cross-sections were cut and mounted onto polylysine-coated glass slides. Slides were heated for 2 h at 58 °C prior to deparaffinization and rehydration by a series of rinses in xylene (3 times for 1 min), 100% ethanol (3 times for 1 min), 95% ethanol (3 times for 1 min), 80% ethanol (1 time for 1 min) and water (1 time for 1 min). Antigen retrieval was performed by immersing the slides in 10 mM citrate, 0.05 % Tween-20, pH 6.0 buffer at 90 °C–100 °C for 10 min. After cooling to RT for 10 min in DPBS, slides were blocked with buffer consisting of 10% normal goat serum in DPBS (NGS, Invitrogen cat. # 50062z) for 1 h at RT. Slides were next incubated with a rabbit anti-human collagen IV polyclonal primary antibody (Invitrogen Cat. # PA5-95188, diluted to 5 μg/mL in buffer consisting of 10% NGS plus 0.1% Tween-20) for 1 h at RT. The samples were then rinsed 3 times for 5 min each with 10% NGS before incubation with goat anti-rabbit IgG H&L Alexa Fluor®555 (1/1,000 dilution, abcam cat. Ab150078) plus Hoechst 33,342 trihydrochloride trihydrate (1/10,000 dilution, Invitrogen cat. #H3570) for 1 h at RT. Samples were then rinsed 3 times for 5 min each with 10% NGS before applying Anti-Fade Fluorescence Mounting Medium (abcam cat. 104135) and application of cover slips. Images were obtained on an Olympus IX51 Fluorescence microscope equipped with CellSens imaging and analysis software. Note: IF analyses were kept to a minimum to avoid use of animal-derived antibodies. Development of IF methods that do not rely on animal-derived antibodies and reagents remains as a major goal for future work.

### 2.10 Transmission electron microscopy (TEM)

Assessment of basement membrane ultrastructure at the dermal-epidermal junction (interstitial collagen fibers, lamina densa, hemidesmosomes and related structures) and ultrastructural elements of the stratum corneum (lamellar bodies, intercorneocyte lamellar lipid sheets and corneodesmosomes) were conducted by TEM.

**S**pecimens were fixed overnight at 4 °C in a mixture of 1.25% formaldehyde, 2.5% glutaraldehyde, and 0.03% picric acid in 0.1 M sodium cacodylate buffer, pH 7.4. Fixed tissues were washed with 0.1 M sodium cacodylate buffer and post-fixed with 1% osmium tetroxide/1.5% potassium ferrocyanide for 2 h at RT. Tissues were then washed 2x in water, 1x in 50 mM Maleate buffer (MB), pH 5.15, and incubated in 1% uranyl acetate in MB for 1 h. After another wash in MB and 2x in ddH_2_0, tissues were dehydrated though a series of ethanol (50%, 70%, 95%, (2x) 100%) for 15 min per solution. Dehydrated tissues were put in propylene oxide for 1 h and infiltrated overnight at 4 °C in a 1:1 mixture of propylene oxide and TAAB Epon. The tissues were embedded in TAAB Epon the following day.

For optimal visualization of multilayered lamellar lipid structures, some tissues were post-fixed in aqueous 0.5% ruthenium tetroxide for 1 h in darkness at RT in place of 1% osmium tetroxide/1.5% potassium ferrocyanide and 1% uranyl acetate, as described by Madison et al. (1987) and van der Meulen et al. (1996). Tissues were dehydrated 2x for 30 min in 70% ethanol and infiltrated in a series of LX-112 resin/70% ethanol mixtures: 1:3 for 30 min, 1:1 overnight at 4 °C, and 3:1 for 30 min the next day. Tissues were then embedded in 100% LX-112 resin.

All samples were polymerized in a 60 °C oven for 48 h. 70 nm ultrathin sections were cut on a Reichert Ultracut-S microtome, mounted onto copper grids, and stained with 0.5% uranyl acetate followed by 0.2% lead citrate. Grids were examined in a TecnaiG^2^ Spirit BioTWIN Transmission Electron Microscope and images were recorded with an AMT *NanoSprint43* CCD camera. Quantitative measurements were obtained with CellSens imaging and analysis software (Olympus).

TAAB Epon was obtained from TAAB Laboratories Equipment Ltd. (https://taab.co.uk). All chemicals (except the TAAB Epon) are from Electron Microscopy Sciences (www.emsdiasum.com).

### 2.11 Viability assays

FT-Skin model viability was measured using MTT or WST-8 reagents. Both of these tetrazolium reagents are reduced by dehydrogenase activities in viable cells to produce colored formazan dyes. The amount of the formazan dye produced is directly proportional to the number/activity of living cells (Mosmann, 1983; Ishiyama, et al., 1997).

MTT powder was dissolved in high-glucose DMEM (1.0 mg/mL) and sterile filtered. FT-Skin models were incubated with 0.8 mL of MTT solution in the reservoir wells below each FT-Skin tissue (basolateral exposure only) for 3 h at 37 °C as specified by OECD Test Guideline No. 439 for *in vitro* skin irritation (OECD, 2025). The tissues were then gently blotted from below to remove excess MTT reagent and transferred to fresh 24-well plates, where they were completely submerged with 2.0 mL of 100% isopropanol to extract the formazan dye from the tissue. The plates were sealed in plastic zip-lock bags and extracted overnight at 4 °C. The plates were then gently agitated on a plate shaker for at least 2 h at RT. After extraction, the extractant from the apical side of each insert was combined with the extractant in the well beneath the same insert, and the combined extract for each well was mixed. Then, 200 μL of extractant solution from each well was transferred to a 96-well plate (n = 2 technical replicates per well), and the optical density was measured at 570 nm with 650 nm subtraction. Isopropanol was used to determine background absorbance. The MTT viability assay was utilized in conjunction with the functional barrier assessment (ET50 Assays) as described in Section 2.12 below.

WST-8 produces a soluble yellow formazan that does not require extraction. The WST-8 reagent stock solution was diluted 1/10 in high-glucose DMEM (WST-8 working solution). FT-Skin models were incubated with 0.8 mL of WST-8 working solution in the reservoir wells below each FT-Skin tissue (basolateral exposure only) for 2 h at 37 °C. Then, 100 μL of the solution from each well was transferred to a 96-well plate (n = 2 technical references per well), and the optical density was measured at 450 nm. WST-8 buffer was added to two wells to obtain the background value.

Because the WST-8 assay reagent is non-toxic and does not require a destructive extraction step, tissues subjected to the assay can be returned to culture for additional experimentation or utilized for additional analyses such as histological evaluation.

MTT and WST-8 assay data were used to assess viability of tissues, evaluated as a percent of negative control treated samples. The percent viability was calculated as follows, using background (blank) subtracted OD values:

$$\%\;Viability=\frac{OD\;of\;Test\;Article}{OD\;of\;Negative\;Control}\times 100$$

### 2.12 Functional barrier assay

Assessment of the FT-Skin model stratum corneum barrier strength was accomplished by a time-to-toxicity (ET50) assay following topical application of 1.0% Triton X-100 solution in tissue culture grade water following a procedure currently specified by OECD TG 439 for *in vitro* skin irritation testing (OECD, 2025).^1^ This assay allows direct comparison of barrier strength with commercial skin models that currently utilize this assay for routine measurement of their skin model barrier as a QC release criterion (OECD TG 439, 2025). To perform the Triton X-100 ET50 assay, 12 FT-Skin tissues were transferred to a fresh 24-well plate containing 800 μL/well of EDM. No ALI lifter was used for this step. When using a 24-well HTS format, the entire plate containing all 24 tissues must be transferred, even though only 12 tissues will be used for the assay. Wells for the remaining 12 tissue must also contain 800 μL of EDM. The additional tissues may be used for other purposes (e.g., other experimental exposures, or removed for gene expression analysis or histological assessment) either before or after the performance of the ET50 assay. After transferring the tissues to the fresh plate, 50 μL of the Triton X-100 solution was dispensed onto the apical surface of n = 2 tissues per timepoint at four timepoints (16, 8, 4 and 1 h). N = 2 tissues were exposed to vehicle (H_2_O) at the 8-h timepoint, and n = 2 tissues were left untreated as controls. The assay was initiated by dosing the tissues for the longest timepoint first (n = 2 tissues, 16-h timepoint), and placing the plate into a 37 °C/5% CO_2_ incubator. The plate was sequentially removed and returned to the incubator for the remaining timepoint exposures such that all exposures were completed at the same time. After the exposure period, the Triton X-100 was aspirated, and the apical side of each tissue was rinsed 3 times with 300 μL of DPBS, before measuring the tissue viability for all 12 tissues using the MTT assay. The percent viability of the tissues at each timepoint (compared to the H_2_O vehicle control tissues) was plotted vs. exposure time, and the time required to reduce tissue viability to 50% of control (ET50) was calculated from the curve.

### 2.13 Statistics

Data from replicate biological samples and replicate independent experiments were expressed as mean +/- standard deviations. Coefficients of variation are defined as the ratio of the standard deviation to the mean and expressed as percentage. ANOVA analysis with Tukey *post hoc* HSD test (Astasta.com) was utilized for statistical analysis of 3 or more data groups. Statistical comparison of 2 data groups was performed using Student’s t-test. The normality of sample distributions was assessed using the Shapiro-Wilk test. The threshold for determining statistical significance was p < 0.05.

## 3 Results

### 3.1 Medium and protocol development for production of FT-skin tissues on electrospun scaffolds

Initial experiments developed media formulations and a general protocol (Figure 3) for production of FT-Skin models on Bio-Spun® PET scaffolds. These media formulations included: 1) DDM consisting of FGM-2 (with 2% FBS) supplemented with 2-phospho-L-ascorbic acid and TGF-β1; 2) ESM consisting of Medium 154 (M154) supplemented with HKGS and 2-phospho-L-ascorbic acid; and 3) a novel EDM consisting of Medium 154 blended 1:1 with DMEM and additional supplements (see Materials and Methods, and Supplementary Material for complete details).

EDM blends based on several alternate commercially available keratinocyte growth media were also evaluated, but were found to produce poor results (Supplementary Material). However, EpiLife keratinocyte growth medium, a medium also formulated for use with HKGS (ThermoFisher), was not evaluated during the initial experiments.

**FIGURE 5:**
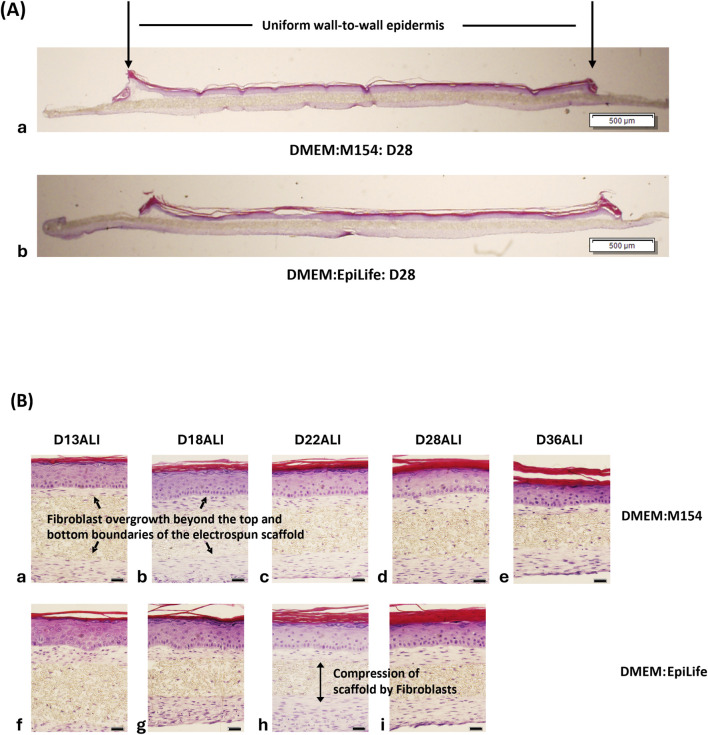
Histologic Evaluation of FT-Skin Tissues Produced from DMEM:M154 vs. DMEM:EpiLife-based EDM. Using optimized DDM and EDM conditions, FT-Skin tissues produced with Medium 154-based ESM and EDM were compared to those produced with EpiLife-based ESM and EDM. **(A)** H&E-stained cross sections at low magnification show uniform coverage of the entire scaffold with no contraction out to 28 days of ALI culture (D28ALI). Scale bar = 500 μm. **(B)** H&E-stained cross sections at high magnification show stable morphology, epidermal thickness and stratum corneum development out to 28 days of ALI culture (D28ALI) for both media conditions. DMEM:M154 tissues maintained epidermal thickness out to D36ALI (longest timepoint evaluated for DMEM:EpiLife was D28ALI). Scale bars = 20 μm.

Additional experiments using the general protocol outlined in Figure 3 were subsequently conducted to extensively evaluate the morphology and lifespan of FT-Skin tissues produced with either M154- or EpiLife-based media. Using Bio-Spun® PET scaffold inserts, both the M154- and EpiLife-based media formulations were found to produce FT-Skin tissues with uniform differentiated epithelia composed of 6–8 stratified cell layers. *In vivo*-like epidermal morphology consisting of basal, spinous, granular and orthokeratinized stratum corneum layers were maintained for as long as 36 days of ALI culture (Figure 5).

It is noteworthy that the dermal development conditions utilized in the above experiments, wherein NHDF were seeded at a density of 150,000 cells/cm^2^, and subsequently cultured for a period of 2 weeks prior to seeding of NHEK, produced a significant overgrowth of fibroblasts and matrix outside of the margins of the Bio-Spun® scaffold. This can be observed on the underside of the scaffold, as well as at the interface between the dermal and epidermal components of the tissue model (Figure 5, Supplementary Figure S4). Furthermore, abundant NHDF overgrowth caused a marked compression of the electrospun scaffold in the vertical direction (Figure 5; Supplementary Figure S4). While the dermal growth outside of the scaffold was not detrimental in terms of overall epidermal structure and morphology, it may be more than necessary or desired depending upon the potential end use of the models. Subsequent experiments that will be discussed in Section 3.4 below found that the amount of fibroblast overgrowth can be controlled by adjusting the initial seeding density of the NHDF and the time of dermal development in the presence of TGF-β1 prior to addition of the NHEK.

Biodegradable PDLGA/PLLA bilayer scaffolds were also evaluated for ability to support FT-Skin model development. DDM, Medium 154-based ESM and EDM, and the production protocol as described above were utilized. Histological evaluation showed that the PDLGA/PLLA bi-layer scaffold also produced a well-developed full-thickness skin construct (Supplementary Figure S6). The PDLGA/PLLA-based FT-Skin models were not extensively characterized beyond these initial experiments. Suitability for long-term *in vitro* assays remains to be determined. However, the biodegradable nature of these scaffolds may make them useful for *in vivo* clinical wound healing applications where biodegradation and ultimate disappearance is desired.

### 3.2 Evaluation of FT-skin tissue development with multiple fibroblast/keratinocyte donor pairs

The general FT-Skin model development protocol and medium formulations described above in Section 3.1 were developed using a random pair of neonatal NHDF and HEKn obtained from Lonza and Gibco, respectively. To verify the robustness of the protocols and media formulations, testing was conducted with additional fibroblast/keratinocyte pairs. Including the initial NHDF and NHEK donor pair, 4 NHDF and 4 HEKn/NHEK donors were tested in various pairings, some of which were matched from the same donor, and some where NHDF and NHEK were from different donors. Complete donor information for all NHDF and HEKn/NHEK are provided in Supplementary Table S1. Histological results after 14 days of ALI culture (D14ALI) show that well-developed FT-Skin models were obtained from all of the donor pairs. Epidermal thickness, keratinocyte morphology and stratum corneum development were generally comparable for all donor pairs, although some differences in fibroblast growth and overall epidermal thickness were apparent (Supplementary Figure S7).

### 3.3 Replacement of FBS in NHDF growth medium and dermal development medium (DDM)

Experiments were conducted to evaluate the ability of HPL to maintain NHDF proliferation and function through multiple serial passages (see Section 2.3.1.2). It was determined that 1.0%–2.5% HPL produced equal or better proliferation than the standard 2.0% FBS-containing medium. Proliferation significantly slowed with 0.5% and was very much diminished with 0.25% HPL (Table 1). HPL concentrations of 1.0%–2.0% were subsequently utilized in place of FBS for NHDF expansions and DDM used in experiments described in Sections 3.4–3.6.

**TABLE 1:** Growth of normal human dermal fibroblasts (NHDF) cultured in fibroblast growth medium (FGM-2) supplemented with human platelet lysate (HPL) as a replacement for fetal bovine serum (FBS).

NHDF (donor 19TL149597)	Fold expansion: Culture time	
Conditions	Passage 4	Passage 5
FGM with 2% FBS (positive control)	13.4 fold: 5 days (doubling time = 32.05 h)	13.40 fold: 3 days (doubling time = 19.23 h)
FGM without FBS or HPL (negative control)	1.1 fold: 10 days (doubling time = 1,744 h)	Not done
FGM without FBS, with 2.5% HPL	14.50 fold: 5 days (doubling time = 31.10 h)	19.80 fold: 3 days (doubling time = 16.72 h)
FGM without FBS, with 1.0% HPL	14.40 fold: 5 days (doubling time = 31.18 h)	12.30 fold: 3 days (doubling time = 19.89 h)
FGM without FBS, with 0.5% HPL	12.80 fold: 5 days (doubling time = 32.63 h)	12.80 fold: 4 days (doubling time = 26.10 h)
FGM without FBS, with 0.25% HPL	5.25 fold: 6 days (doubling time = 60.19 h)	7.67 fold: 4 days (doubling time = 32.67 h)

Cryopreserved passage-3 NHDF, were serially passaged in FGM-2 containing the indicated concentrations of HPL in place of FBS. Standard FGM-2 with 2.0% FBS, and FGM-2 without FBS or HPL served as the positive and negative control conditions, respectively. HPL was tested at concentrations of 2.5%, 1.0%, 0.5%, and 0.25%. Cells were seeded at an initial density of 6,667 cells/cm^2^, and counted at each passage prior to reseeding at the same density. Cells were harvested and cryopreserved at passage 5, with the exception of the negative control condition which was not continued beyond passage 4.

### 3.4 Development and evaluation of an optimized 3-week FT-skin model production protocol

**FIGURE 6:**
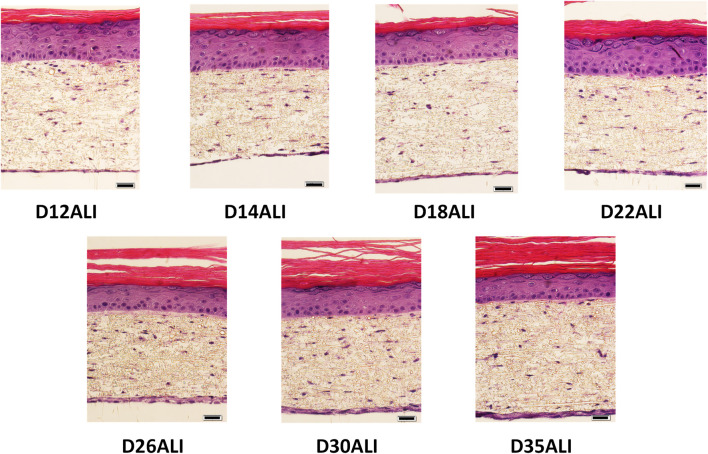
Lifespan of FT-Skin Tissues Produced with 3-week production Protocol. FT-Skin tissues were produced using the 24-well individual Bio-Spun® PET insert format with direct seeding of NHDF and HEKn, and the 3-week production protocol. FT-Skin tissue samples were periodically fixed for analysis out to 35 days of ALI culture. H&E-stained cross sections demonstrate maintenance of good morphological structure with stable thickness of the viable epithelial layer out to day 35 of ALI culture, with only slight thinning of the epidermis noted only at day 35. Scale bars = 20 μm.

Through an iterative process, the effects of NHDF seeding density and duration of the dermal culture period were further evaluated. It was found that the amount of fibroblast overgrowth outside of the electrospun scaffold can be controlled by adjusting the initial seeding density of the NHDF and the time of dermal development in the presence of TGF-β1 prior to addition of the NHEK. Ultimately, the dermal development time was reduced to as low as 3 days, resulting in a reduction of the FT-Skin model development time to only 3 weeks (Section 2.7.2; Figure 4). These conditions also eliminated the excessive fibroblast overgrowth seen on the underside of the scaffold and at the interface between the dermal and epidermal components (Supplementary Figure S4, Figure 6).

The morphological lifespan of the FT-Skin model produced using the 3-week protocol was evaluated using the 24-well individual Bio-Spun® PET insert Format, NHDF donor 19TL149597 at final passage P6, and HEKn donor 2491219 at final passage P4. DDM was formulated with HPL in place of FBS. ESM and EDM utilized Medium 154 and HKGS. FT-Skin tissue samples were periodically fixed for histological analysis out to 35 days of ALI culture. The FT-Skin tissues maintained good morphological structure with stable thickness of the viable epithelial layer out to day 35 of ALI culture, with slight thinning of the epidermis noted only at day 35 (Figure 6). These results are similar to those obtained previously with the General Production Protocol, wherein a 2-week dermal culture period and the 24-well-HTS Bio-Spun® PET format were used (Figure 5). The 3-week protocol was adopted for use in subsequent experiments described below in Sections 3.4–3.6.

### 3.5 FT-skin model protocol transfer and assessment of functional barrier

In order to test the transferability and reproducibility of the FT-Skin model production protocol, FT-Skin models were produced in an independent contract research laboratory (the Institute for *In Vitro* Sciences, IIVS) following the 3-week protocol as described in Section 2.7.2, Figure 4. Tissue morphology and baseline viability were determined by histological analysis and WST-8 metabolism assays, as described in Sections 2.8, 2.11, respectively. A time-to-toxicity (ET50) barrier assessment assay that is commonly used as a quality control test and a basic component of commonly accepted regulatory assays was performed as described in Section 2.12.

Parallel FT-Skin model tissue lots were produced at BioSurfaces (Ashland, MA) and IIVS (Gaithersburg, MD) using the same lots of Bio-Spun® PET scaffold inserts, DDM, ESM, EDM, cryopreserved NHDF (donor 19TL149597, P6) and HEKn (donor 2491219, P4). DDM was formulated with HPL in place of FBS, and ESM and EDM culture media were both formulated with Medium 154 and HKGS. All materials, media and cell lots were prepared at BioSurfaces and shipped to IIVS by overnight courier. IIVS staff received a previous ½ day of in-person training at IIVS and further 1 ½ h training by zoom conference prior to performing the production runs. The cryopreserved NHDF and HEKn were thawed and directly seeded without further expansion as described in Section 2.7.2.

Well-to-well baseline viability was assessed on n = 6 random tissues from 3 independent production runs from both laboratories using the WST-8 assay. Results show a low coefficient of variation (cv) ranging between 5.87% and 11.64%, and good concordance between laboratories (Table 2). One-way ANOVA showed that run #2 was significantly different from runs #s 1 and 3 (p < 0.01). No statistically significant difference was observed between run #s 1 and 3 (p = 0.55). No overall statistically significant difference was observed between laboratories (2-tailed t-test, p = 0.67).

**TABLE 2:** Inter- and intra-laboratory reproducibility of baseline FT-Skin tissue viability (WST-8 Assay).

Run number	Average OD ± standard deviation of n = 6 random FT-Skin tissues	
	BioSurfaces	IIVS
Run #1	1.44 +/- 0.08	1.60 +/- 0.11
Run #2	1.24 +/- 0.12	1.10 +/- 0.13
Run #3	1.65 +/- 0.18	1.22 +/- 0.09

Parallel tissue lots were produced at BioSurfaces (Ashland, MA) and IIVS (Gaithersburg, MD) using the same lots of Bio-Spun® scaffold inserts, cryopreserved NHDF and HEKn, and DDM, ESM and EDM culture media. All materials were prepared at BioSurfaces and shipped to IIVS by overnight courier. Well-to-well baseline viability was assessed on n = 6 random tissues from 3 independent production runs from both laboratories using the WST-8 assay. Run #2 is significantly different then run #1 and run #3: One-way ANOVA p < 0.01. Runs # 1 and #3 are not significantly different: One-way ANOVA p = 0.55. No significant overall difference between laboratories was observed: Two-tailed t-test p = 0.67.

**FIGURE 7:**
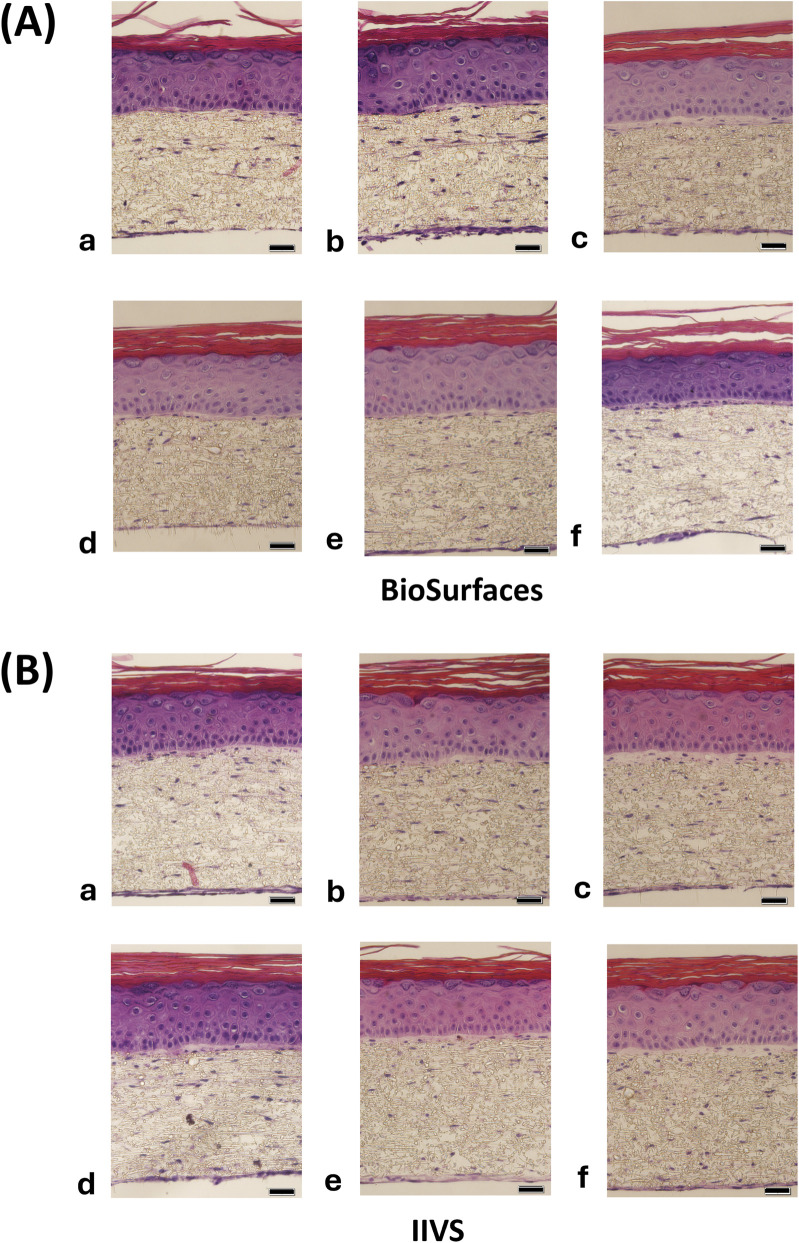
Intra- and Inter-Laboratory Reproducibility of FT-Skin Model Histology Produced with the 3-week production Protocol. FT-Skin models were produced concurrently at BioSurfaces (Ashland, MA) and IIVS (Gaithersburg, MD) laboratories, using the same lots of cells, media and scaffold inserts. N = 6 random FT-Skin tissues **(a-f)** were evaluated from each laboratory. H&E-stained cross sections show comparable morphology, epidermal thickness and stratum corneum development at 14 days of ALI culture (D14ALI). Scale bars = 20 μm.

Histological assessment shows that the morphology of FT-Skin tissues produced by BioSurfaces and IIVS was virtually indistinguishable between laboratories. A robust viable epithelium with similar thickness, showing well-developed basal, spinous and granulosum layers was produced by both laboratories. Stratum corneum development between the 2 sets of FT-Skin tissues was also similar (Figure 7).

The Triton X-100 (TX-100) ET50 assay as described in Section 2.12 was also conducted to assess the functional barrier of the FT-Skin tissues produced by the 2 laboratories in 3 independent production runs. The barrier of the FT-Skin tissues was found to be highly reproducible within and between laboratories (Table 3). The observed average ET50 of 12.51 +/− 0.37 h (cv = 2.97 %) demonstrated a very robust stratum corneum barrier of the models. No statistically significant differences were found between production runs (1-way ANOVA p = 0.30) or laboratories (2-tailed t-test p = 0.052). In addition to the 3 independent comparisons conducted with HEKn donor 2491219 that were conducted in both laboratories, an additional evaluation was conducted using HEKn donor 2825951 at BioSurfaces only. The ET50 of FT-Skin tissues produced from donor 2825951 closely matched those of donor 2491219 (Table 3).

**TABLE 3:** Inter- and intra-laboratory reproducibility of FT-Skin tissue barrier (Triton X-100 ET50 assay).

Run number	1.0% triton X-100 ET50 (h)	
	BioSurfaces	IIVS
Run #1 (2,491,219 NHEK)	12.68	12.21
Run #2 (2,491,219 NHEK)	13.14	12.55
Run #3 (2,491,219 NHEK)	12.36	12.12
Run #1 (2,825,951 NHEK)	12.80	​

Parallel tissue lots were produced at BioSurfaces (Ashland, MA) and IIVS (Gaithersburg, MD) using the same lots of Bio-Spun® scaffold inserts, cryopreserved NHDF and HEKn, and DDM, ESM and EDM culture media. All materials were prepared at BioSurfaces and shipped to IIVS by overnight courier. The Triton X-100 (TX-100) ET50 assay as described in Section 2.11 was also conducted to assess the functional barrier of the FT-skin tissues produced by the 2 laboratories in 3 independent production runs. The overall average ET50 for 2,491,219 NHEK-based FT-skin models was 12.51 +/- 0.37 h (cv = 2.97%). There are no significant differences between production runs (one-way ANOVA p = 0.30) or laboratories (two-tailed t-test p = 0.052).

### 3.6 Elimination of bovine pituitary extract (BPE) from epidermal submersion medium (ESM) and epidermal differentiation medium (EDM)

Experiments were conducted in a 24-well HTS format following the 3-week protocol described above in Section 2.7.2 using NHDF (donor 19TL149597, final passage P6) and HEKn (donor 2491219, final passage P4). DDM was prepared with HPL in place of FBS. ESM was prepared from either M154 or EpiLife medium, and EDM was prepared using either DMEM:M154 or DMEM:EpiLife as the base formulations. In both cases, the HKGS supplement was replaced by Supplement S7. Alternatively, the BPE component was left out of the HKGS kit for some experiments. All other components of the ESM and EDM media as described in Sections 2.3.2 and 2.3.3 were unchanged.

**FIGURE 8:**
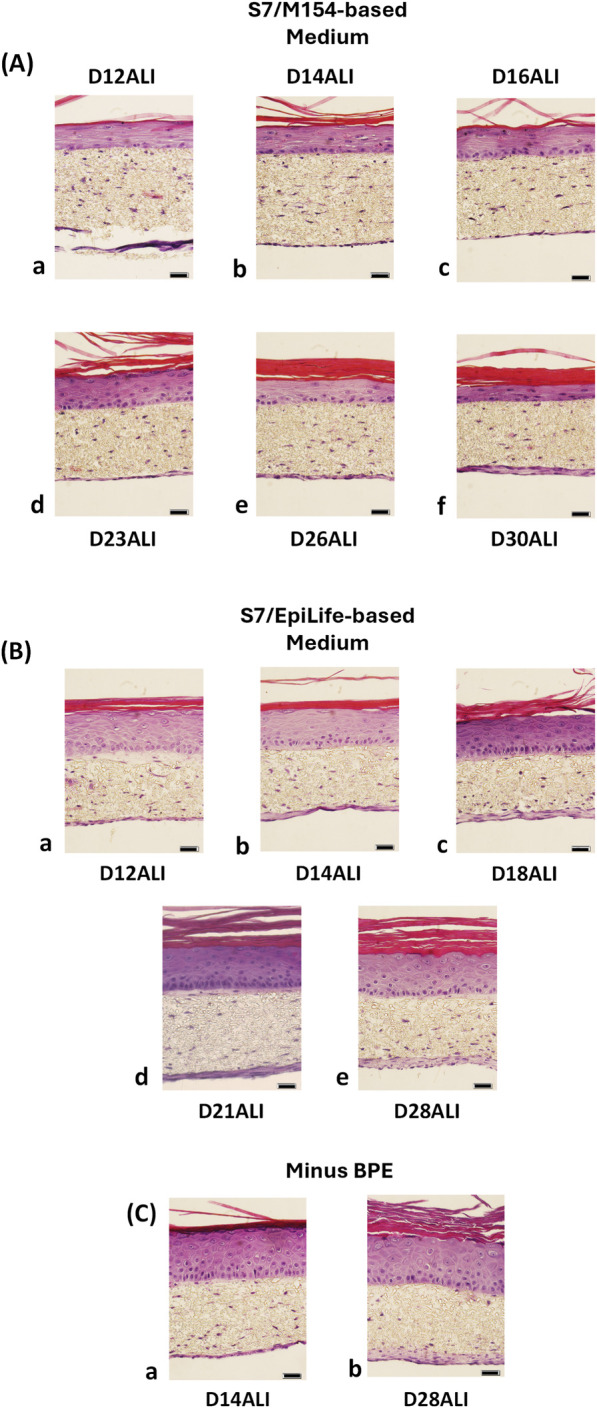
Histological Evaluation of FT-Skin Tissue Produced with HKGS Replaced by Supplement S7, or Removal of BPE from the Culture Medium. Experiments were conducted using 150 μm thick PET scaffolds in a 24-well HTS format following the 3-week production protocol. DDM was prepared with HPL in place of FBS. ESM was prepared from either M154 or EpiLife medium, and EDM was prepared using either DMEM:M154 or DMEM:EpiLife as the base formulations. In both cases, the HKGS supplement was replaced by Supplement S7. Alternatively, the BPE component was left out of the HKGS kit for some experiments. All other components of the ESM and EDM media were unchanged. H&E-stained cross sections of FT-Skin tissues produced with HKGS replaced by Supplement S7 using **(A)** DMEM/M154-based EDM, **(B)** DMEM/EpiLife-based EDM**,** and **(C)** HKGS-based EDM without BPE. DMEM:M154-based EDM produced tissues with more flattened keratinocyte morphology, and the overall thickness of the epithelium was decreased by day 28 ALI compared to DMEM:EpiLife-based EDM. Removal of BPE from HKGS-based EDM with no additional modifications also produced FT-Skin tissue with well-developed epithelial morphology to day 28 ALI. Scale bars = 20 μm.

Histological evaluation showed that replacement of HKGS with Supplement S7 in either M154- or EpiLife-based ESM and EDM produced FT-Skin tissues with well-developed epidermis and stratum corneum that was maintained for at least 28–30 days of ALI culture. DMEM:M154-based EDM produced tissues with more flattened keratinocyte morphology, and the overall thickness of the epithelium was decreased by day 28 ALI compared to DMEM:EpiLife-based EDM (Figures 8A,B). Removal of BPE from HKGS-based EDM with no additional modifications also produced FT-Skin tissue with well-developed epithelial morphology to day 28 ALI (Figure 8C).

### 3.7 Effect of culture and medium conditions on maintenance of epidermal thickness over time

During the current project, a set of 5 independent experiments assessed how the length of the dermal culture period and specific components of the ESM and EDM media formulations affected morphological integrity and epidermal thickness over the course of up to 36 days of ALI culture. The five experimental conditions included:

- General protocol with 14-day dermal culture (G14D) using FBS-based DDM and Medium 154/HKGS-based ESM and EDM (G14D/FBS:M154/HKGS; histology shown in Figure 5)
- General protocol with 14-day dermal culture (G14D) using FBS-based DDM and EpiLife/HKGS-based ESM and EDM (G14D/FBS:EpiLife/HKGS; histology shown in Figure 5)
- 3-Week protocol with 3-day dermal culture (3W3D) using HPL-based DDM and Medium 154/HKGS-based ESM and EDM (3W3D/HPL:M154/HKGS; histology shown in Figure 6)
- 3-Week protocol with 3-day dermal culture (3W3D) using HPL-based DDM and Medium 154/Supplement S7-based ESM and EDM (3W3D/HPL:M154/S7; histology shown in Figure 8A)
- 3-Week protocol with 3-day dermal culture (3W3D) using HPL-based DDM and EpiLife/Supplement S7-based ESM and EDM (3W3D/HPL:EpiLife/S7; histology shown in Figure 8B)

**FIGURE 9:**
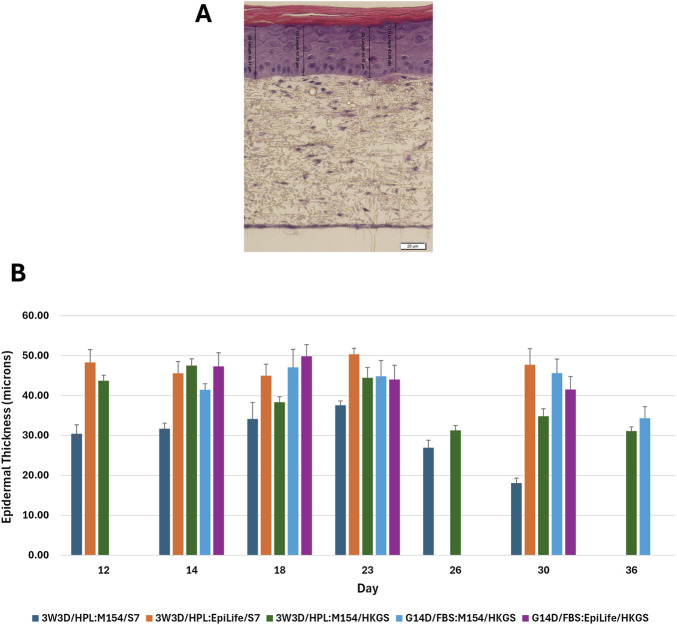
Quantitative Evaluation of Epidermal Thickness Over Time. A set of 5 independent experiments assessed how dermal culture duration as well as ESM and EDM media formulations affected morphological integrity and epidermal thickness over 36 days of ALI culture. **(A)** The thickness of epidermal layers was measured at 4 locations within a representative field of view. **(B)** Thickness values shown are averages +/- standard deviation for each sample. Thickness values shown are averages +/- standard deviation for each sample. 3W3D/HPL:M154/S7 = 3-week protocol with 3-day dermal culture using HPL-based DDM and Medium 154/Supplement S7-based ESM and EDM. 3W3D/HPL:EpiLife/S7 = 3-week protocol with 3-day dermal culture using HPL-based DDM and EpiLife/Supplement S7-based ESM and EDM. 3W3D/HPL:M154/HKGS = 3-Week protocol with 3-day dermal culture using HPL-based DDM and Medium 154/HKGS-based ESM and EDM. G14D/FBS:M154/HKGS = General protocol with 14-day dermal culture using FBS-based DDM and Medium 154/HKGS-based ESM and EDM. G14D/FBS:EpiLife/HKGS = General protocol with 14-day dermal culture using FBS-based DDM and EpiLife/HKGS-based ESM and EDM.

In order to quantify the thickness of the epidermal layer over time for each condition, measurements were made from the histological cross sections at 4 locations within a representative field of view to include what were judged to be the 2 thinnest areas and the 2 thickest areas of each sample. A summary of epidermal thickness values from all 5 experimental data sets are shown in Figure 9 and Supplementary Table S2.

The epidermal thickness was maintained above 30 μm out to at least 30 days for all conditions, with the exception of the 3W3D:M154/S7 condition, which diminished to <20 μm by day 30. However, FT-Skin tissues produced from both 3W3D/HPL and G14/FBS dermal protocols, followed by use of M154/HKGS-based media during epithelial development, maintained epidermal thickness above 30 μm out to at least 36 days. The completely animal-free condition consisting of 3W3D/HPL:EpiLife/S7-based media provided the most stable maintenance of epidermal thickness, which was above 40 μm for all timepoints analyzed for at least 30 days (Figure 9; Supplementary Table S2).

### 3.8 Characterization of stratum corneum barrier and basement membrane ultrastructure by transmission electron microscopy (TEM)

TEM was utilized to confirm development of basement membrane ultra-structures, (interstitial collagen fibers, lamina densa, hemidesmosomes and related structures) and ultrastructural elements of the stratum corneum (lamellar bodies, intercorneocyte lamellar lipid sheets and corneodesmosomes). Samples from FT-Skin tissues produced using the 3-week production protocol described in Section 2.7.2 and Figure 4, with HPL-containing DDM, and either HKGS- or Supplement S7-containing ESM and EDM media were analyzed (duplicate samples each for the HKGS- and Supplement S7-containing conditions). Both the HKGS and Supplement S7 media produced FT-Skin tissues with similar well-developed ultrastructural features.

**FIGURE 10:**
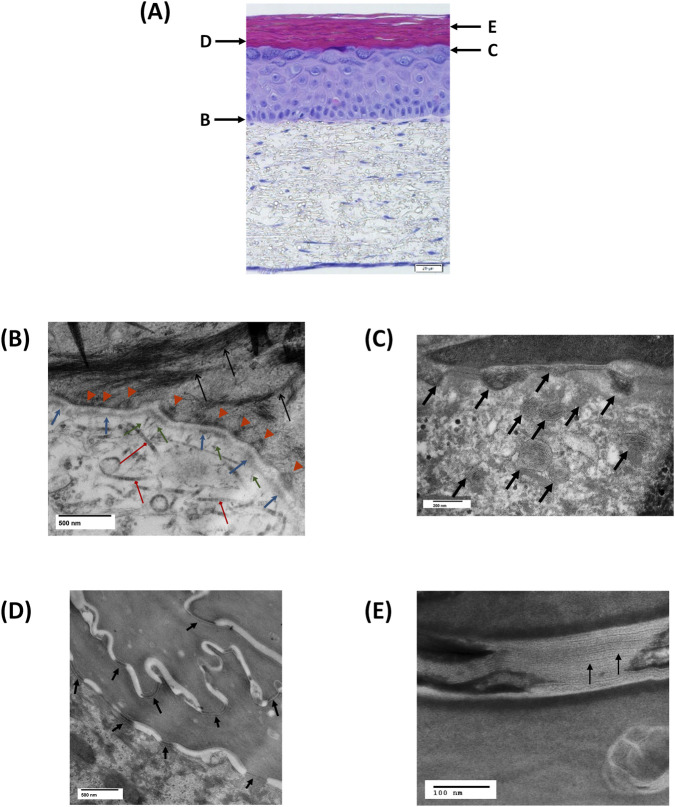
Transmission Electron Microscopy (TEM) of Key Basement Membrane and Stratum Corneum Structural Elements. TEM was utilized to confirm development of basement membrane ultra-structures (interstitial collagen fibers, lamina densa, hemidesmosomes and related structures) and ultrastructural elements of the stratum corneum (lamellar bodies, intercorneocyte lamellar lipid sheets and corneodesmosomes). Representative samples produced using the 3-week FT-Skin model protocol described in Section 2.7.2 using Supplement S7-containing ESM and EDM media are shown **(A)** Specific area of focus for TEMs shown in B-E are depicted by the corresponding arrows. Scale bar = 20 mm. **(B)** Well-developed basement membrane structures including hemidesmosomes (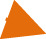), lamina densa (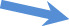), collagen fibers (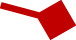), anchoring fibrils (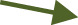) and tonofilaments (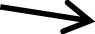). Scale bar = 500 nm **(C)** Abundant numbers of lamellar bodies are present in the upper layers of the stratum granulosum, with secretion into the interface with the stratum corneum (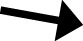). Scale bar = 200 nm **(D)** Corneodesmosomes are prominent at the junction of the granular epidermis-stratum corneum interface and the lower layers of stratum corneum corneocytes (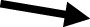). Scale bar = 500 nm **(E)** Contents of the lamellar bodies are assembled into continuous sheets that fill the spaces between stratum corneum corneocytes (
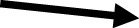
). Scale bar = 100 nm.

TEM micrographs of the basal lamina showed striated collagen fibers and a well-developed basement membrane with lamina densa, anchoring fibrils and hemidesmosomes. Tonofilaments and tonofibrils emanating from hemidesmosomes extend into the cytoplasm of the basal keratinocytes (Figure 10B). The lamina densa thickness was quantified for further comparison between the HKGS- and Supplement S7-based media. Measurements were made using 10 micrographs from each medium condition (5 each from n = 2 samples per condition) with four areas measured from each micrograph (n = 40 total measurements for each condition). The average lamina densa thickness was 61.43 +/− 7.34 nm for HKGS-based media and 63.03 +/− 4.93 nm for Supplement S7-based media. There was no statistically significant difference between the lamina densa thickness of the 2 media conditions (two-tailed t-test, p = 0.18). The number of hemidesmosomes per μm was also assessed for each medium condition. Most micrographs showed at least 2-3 hemidesmosomes per μm for both media conditions.

TEM micrographs of the stratum granulosum/stratum corneum region show abundant lamellar bodies in the upper layers of the stratum granulosum, and secretion of the lipids into the interface with the stratum corneum (Figure 10C). Corneodesmosomes, specialized structures that are unique to properly differentiated epidermis (Haftek, 2015) are also prominent at the junction of the granular epidermis-stratum corneum interface and the lower layers of stratum corneum corneocytes (Figure 10D). Further maturation of the lamellar lipids into inter-corneocyte sheets is important for development of the stratum corneum barrier (Leprince & Simon, (2025). TEM micrographs confirm that contents of the lamellar bodies are assembled into continuous sheets that fill the spaces between stratum corneum corneocytes (Figure 10E).

### 3.9 Immunofluorescence microscopy (IF) of collagen IV deposition and localization

**FIGURE 11:**
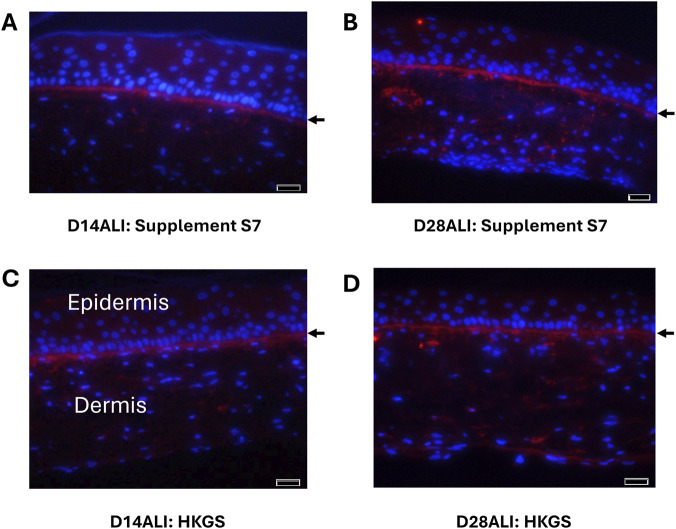
IF Evaluation of Collagen IV Deposition and Localization. Immunofluorescence microscopy was conducted to confirm the presence and localization of collagen IV in the FT-Skin model. Samples of FT-Skin produced using the 3-week production protocol with both the Supplement S7- **(A,B)** and HKGS- based **(C,D)** culture media were evaluated after 14 and 28 days of ALI culture. Collagen IV was confirmed to be abundantly present and specifically localized at the dermal-epidermal junction (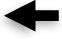) for all conditions. Scale bars = 20 μm.

As further verification of basement membrane development, immunofluorescence microscopy was conducted to confirm the presence and localization of collagen IV in the FT-Skin model. Samples of FT-Skin produced using the 3-week production protocol with HPL-containing DDM and either the HKGS- or Supplement S7-based epidermal culture media were evaluated after 14 and 28 days of ALI culture. Collagen IV was confirmed to be abundantly present and specifically localized at the dermal-epidermal junction (Figure 11). The presence of laminin 332 can also be inferred from these results because assembly of collagen IV at the dermal-epidermal junction (DEJ) will not occur in its absence (Matlin et al., 2017).

## 4 Discussion

### 4.1 Key outcomes of the current work in relation to prior state-of-the-art

The current work has built upon prior advancements from numerous groups to demonstrate the feasibility of producing robust and long-lasting FT-Skin models without the use of animal-derived ECM, FBS, or BPE, the major animal-derived components that are commonly utilized in many current *in vitro* FT-Skin model production processes. This was achieved by combining electrospun scaffolds and tissue cultureware with novel medium formulations, culture techniques and production protocols. Key aspects of the current work in relation to prior art are discussed in detail below.

#### 4.1.1 Elimination of animal-derived collagen and FBS for production of dermal components

Elimination of the need for animal-derived ECM and FBS in production of the FT-Skin models was achieved by using commercially available electrospun scaffold inserts as a structural component of the dermis, in combination with a dermal medium formulation in which FBS was replaced with HPL. Addition of TGF-β1 was also found to be critical for reliably robust dermal development.

Electrospun scaffolds have previously been utilized for both *in vivo* (Jun, et al., 2018; Imaichi-Kobayashi, et al., 2023) and *in vitro* tissue engineering applications (Malakpour-Permlid, et al., 2021) including *in vitro* skin models (Girard et al., 2024; Weigel, et al., 2022). The PLC-based scaffolds described by Girard et al. (2024) require a complex fabrication process that combines both electrospinning and melt electrowriting. The polyamide 6-based scaffolds described by Weigel et al. involve a fabrication process that incorporates porogens during the spinning process. Scaffolds were manually clamped into crown inserts for producing tissue models (Weigel et al., 2022). However, neither of these approaches have been scaled to commercial production levels to-date. In an alternate approach, commercially available Alvetex scaffolds produced from porous polystyrene (Reprocell, Yokohama, JP), have also been shown to support animal-ECM-free full-thickness skin model production (Zoio, et al., 2021; Costello et al., 2022). However, these previously described skin models still employ FBS to develop the dermal constructs, and typically require more complicated and/or longer production processes.

Primary human fibroblasts generally require culture medium containing FBS to maintain long-term proliferation and function. However, the use of FBS is undesirable due to ethical as well as reproducibility and safety reasons (van der Valk, et al., 2018; Rauch, et al., 2011; Weber and Wagner, 2021; Weber et al., 2025). Human platelet lysate (HPL) has been reported as an effective FBS replacement for culture of mesenchymal stem cells (Kandoi, et al., 2018; Karadjian et al., 2020), as well as engineered skin for clinical applications (Banakh, et al., 2023). HPL was therefore tested as a FBS replacement in media utilized for development of the Bio-Spun® FT-Skin model.

Banakh et al. (2023) showed that HPL could support limited serial proliferation of NHDF as well as produce a fibroblast-populated hydrogel that supported epidermal development out to 8 days. The work reported here confirms and significantly expands on those original findings to demonstrate successful use of HPL for serial passage of NHDF and development of long-lasting dermal constructs. In addition to HPL, the dermal development medium utilized in the current work also made use of TGF-β1 to promote ECM production. TGF-β1 is a well-known inducer of ECM production, and has been previously used to enhance ECM production in *in vitro* tumor models based on electrospun PCL scaffolds (Malakpour-Permlid, et al., 2021) and *in vitro* skin models based on porous polystyrene scaffolds (Costello, et al., 2022).

Production, localization and assembly of specific ECM components and structures is essential for guiding the proper development, function and long-term homeostasis of full-thickness skin. Fibroblasts have been previously shown to secrete ECM components including type I and III collagens, integrin ligands including fibronectin and laminins, glycosaminoglycans, proteoglycans, fibrillins and elastin (Tracy, et al., 2016; Malakpour-Permlid, et al., 2021; Huang, et al., 2022), as well as non-structural matricellular proteins (Bornstein, 2009). The work of Malakpour-Permlid, et al. (2021), Weigel, et al. (2022), Zoio et al. (2021) and others has previously characterized ECM components produced from dermal fibroblasts cultured on electrospun or polystyrene scaffolds. TGF-β1is known to be a potent inducer of these ECM components by fibroblasts (Hinz, 2015).

In the current work, Van Gieson’s stain was utilized to show the production of collagen, and TEM and IF was utilized to demonstrate the presence of specific sub-cellular structures that are known to be composed of various aforementioned ECM protein components. The TEM and IF characterizations not only demonstrate the presence of the various ECM proteins, but additionally confirm that the components have properly localized and assembled into functional sub-cellular structures such as collagen fibers (collagens type I and III), lamina densa (collagen type IV, laminin), anchoring fibrils (collagen type VII), hemidesmosomes (various integrins including α6β4 and others), etc., (Breitkreutz et al., 2013; Hayden et al., 2009; Jayadev, et al., 2019). Importantly, these structures are shown to be present in FT-Skin tissues produced using the 3-week culture protocol with HPL and Supplement S7 in place of FBS and BPE (Figure 10).

#### 4.1.2 Animal product-free epidermal differentiation medium (EDM) formulations

Previously described EDM formulations have been based on standard keratinocyte growth media that are modified with elevated calcium levels, ascorbic acid and growth factors such as KGF (Rosdy and Clauss, 1990; Weigel et al., 2022; Zoio et al., 2021). Alternatively, blends of DMEM and Ham’s F12 medium supplemented with ascorbic acid have also been described (Gangatirkar et al., 2007; Jäger et al., 2024). In both cases, BPE and/or FBS are also generally required. Although the beneficial effect of further supplementation with barrier lipid precursors has been demonstrated by Boyce and Williams (1993) and Ponec et al. (1997), most recently described EDM formulation do not include these components.

The current work developed novel EDM formulations consisting of a 50:50 blend of DMEM with either Medium154 or EpiLife medium. The formulations also included HKGS, ascorbic acid, barrier lipid precursors, isoproterenol (cAMP inducer) and GM 6001 (MMP inhibitor). When Supplement S7 is used in place of HKGS, or BPE is omitted from the HKGS, an entirely animal product-free FT-Skin model is possible. This will require substitution of BSA with HSA and bovine transferrin in the HKGS to be replaced with human transferrin, both of which are commercially available, albeit, with higher costs. This novel EDM formulation has been shown to produce FT-Skin models with well-developed morphology that is typical of normal healthy epidermis, consisting of basal, spinous, granular and normal orthokeratinized stratum corneum layers (Zingkou et al., 2022). Additionally, FT-Skin tissues produced with the EDM show improved lifespan and barrier (discussed further below).

#### 4.1.3 ALI insert lifters

Most current commercially available tissue culture inserts and associated cultureware plates have a limited volume capacity for tissue culture medium, and were not designed to accommodate ALI culture. The current work made use of commercially available adaptors which serve to lift the scaffold inserts higher within the plate wells so that larger volumes of tissue culture medium can be accommodated. The use of ALI lifters allows less handling, extended feeding time, and eliminates the need for weekend feedings.

#### 4.1.4 Direct seeding of NHDF and NHEK without final expansion

Protocols utilized to produce FT-Skin tissues typically involve a final expansion and harvest of both NHDF and HEkn/NHEK prior to seeding onto the scaffolds. However, this final expansion step introduces additional complexity, time and variability into the production process, and can cause difficulty if either the NHDF or HEKn/NHEK expansions do not progress as expected. For example, either cell type may grow slower or faster than expected, or a harvest may not produce the expected yield of cells. It is therefore desirable, especially for large-scale or continuous routine production, to eliminate these final expansion steps. The current work implemented protocols for xeno-free cryopreservation of both NHDF and HEKn/NHEK at high cell concentrations to allow for seeding of the cells directly from cryopreservation without the need for the final expansion step. This simplifies and shortens the overall production cycle, and eliminates a major source of variability and potential problems, especially for continuous routine production scenarios.

Implementation of this aspect of the production process requires an initial investment of time to prepare the cells in advance, and generally requires a significant scale-up of cell production to be beneficial. However, the benefits can be very significant. Ideally, large batches of cryopreserved cells are produced and pre-qualified so that each freeze-lot will be used for multiple tissue production lots. Using the same lots of pre-qualified cryopreserved NHDF and HEKn/NHEK over multiple tissue production runs both streamlines and simplifies the routine production process, and eliminates a major source of variability. The larger the final freeze-lots of cryopreserved cells are, the greater the ultimate benefits will be.

### 4.2 Functional benefits provided by the current production protocols and FT-skin model

Aside from the previously described benefits of animal component-free tissue models, several additional important practical and functional benefits were realized from the current work. These include a longer useful lifespan, an improved stratum corneum barrier, faster and easier production process, and high reproducibility and transferability.

#### 4.2.1 Improvement in useful lifespan

Currently available commercial FT-Skin models, such as the Phenion® FT Skin Model (Phenion GmbH and Co. KG) or EpiDermFT™ (MatTek Life Sciences), that are based on proprietary media formulations and protocols, advertise a useful lifespan of 10–14 days after attainment of tissue maturity. More recently, Phenion has developed a proprietary production process and media that reportedly provides a lifespan of up to 50 days (phenion.com). Zoio et al. (2021) has also reported development of a FT-skin model that was maintained for up to 50 days, while Dos Santos et al. (2015) have reportedly maintained FT-skin tissues for up to 120 days.

The FT-Skin models reported in the current work maintained a useful epidermal thickness for at least 36 days of ALI culture. This represents a significant improvement over many current *in vitro* skin models, including most commercially available models. The improved useful lifespan is important for allowing researchers to conduct longer-term experiments that are not possible with many currently available FT-Skin models. These results are also noteworthy in that they have been obtained with late passage NHDF (P6) and NHEK/HEKn (P4), suggesting that further improvements may be obtainable if lower passage cells are used. No attempts were made with regard to optimization of the growth factors and supplement components of the HKGS or Supplement S7. Additional optimization of the media or supplements may provide further improvements in the currently reported FT-Skin model lifespan.

#### 4.2.2 Improvement in stratum corneum barrier

Two structural elements that play critical functional roles in human skin are the stratum corneum and the dermal-epidermal junction basement membrane. The stratum corneum provides the main barrier that protects the body from physical and environmental hazards and water loss. Corneodesmosomes and intercorneocyte lipid lamellae are responsible for stratum corneum cohesion (Ishida-Yamamoto, and Igawa, 2015) and water permeability barrier (Madison et al., 1987; Feingold and Elias, 2014). Epidermal basement membrane structures provide physical attachment of the epidermis to the dermis, and mediate cellular communication between the two components. Cross-talk between the dermis and epidermis is important for regulation of epidermal proliferation, differentiation, wound healing, barrier function, skin diseases, aging/photoaging and immune responses (El Ghalbzouri et al., 2005; Varkey et al., 2014).

Culture conditions utilized in the current work, such as culturing at 50% relative humidity (RH) (Mak et al., 1991; Sun et al., 2015) and media formulations containing lamellar lipid precursors (Boyce and Williams, 1993), 2-phospho-L-ascorbic acid (Ponec, et al., 1997) and proteinase inhibitors (Amano et al., 2001; Iriyama et al., 2018, Iriyama et al., 2019; Varkey et al., 2014), were designed to promote proper development and function of both the stratum corneum and basement membrane structures.

Barrier function of *in vitro* skin models is considered to be an important factor that impacts the relevance and translatability of *in vitro* to *in vivo* results. Aside from stability, reproducibility and lifespan issues, lack of an adequate *in vivo*-like stratum corneum barrier is perhaps the most often cited deficiency of *in vitro* skin models. Media formulations and culture conditions developed in the current work include components such as 2-phospho-L-ascorbic acid and barrier lipid precursors, as well as ALI culture in a 50% RH environment, that were designed to enhance barrier development. TEM confirmed the presence of key basement membrane structures, as well as corneodesmosomes, lamellar bodies, and intercorneocyte deposition of lamellar lipids in the stratum corneum, that are key elements responsible for providing and maintaining epidermal barrier. Functional assessment of the stratum corneum barrier by the ET50 assay confirmed an exceptionally strong barrier compared to other reported skin models. The average ET50 of 12.51 h demonstrated with the current FT-Skin models is the highest reported ET50 of any skin model that we are aware of. The improved stratum corneum barrier is important for providing more accurate and translatable results in *vitro* assessments of epidermal chemical penetration for toxicology and epidermal drug delivery applications.

#### 4.2.3 Streamlined and shortened production process

Production of FT-Skin models is a complex and time-consuming process, typically required up to 5-week per production cycle. In addition, when using traditional tissue cultureware, feeding schedules that often include weekend or even daily feedings are generally required. The long production cycle and need for frequent feeding and handling present formidable obstacles to implementation of long-term routine production, especially if weekly or bi-monthly production cycles are desired, which would necessitate overlapping production runs.

Innovations described above including use of electrospun scaffolds and seeding of NHDF directly from cryopreservation have allowed development of dermal constructs in only 3 days. In addition, use of ALI lifters allows a simplified feeding schedule that requires only 2 or 3 ALI feedings per week, for individual or 24-well HTS insert formats, respectively. Thus, the entire production process can be accomplished in 3 weeks without the necessity for weekend feedings. This faster and simplified production process should greatly improve scalability and facilitate implementation of continuous routine production of FT-Skin models for a wider range of research and production laboratories.

#### 4.2.4 Reproducible and transferable production process

Intra- and inter-lot reproducibility and protocol transferability are also key requirements for successful application of *in vitro* skin models for industrial research applications and regulatory testing. The production process and media formulations reported in the current work were shown to be very robust in terms of working with a broad variety of different NHDF and HEKn/NHEK donor combinations. In addition, the protocols were successfully transferred to an independent laboratory with minimal training. Excellent intra- and inter-lot, as well as inter-laboratory viability, morphological appearance and barrier function were demonstrated. The facile transferability of the FT-Skin tissue model protocols will broaden accessibility and adoption for a wide range of research laboratories. The high reproducibility of the models will meet the stringent requirements for assay validation and use by industrial research laboratories and regulatory agencies.

#### 4.2.5 Open-source production protocols and detailed media formulations

Scalable, reproducible production of *in vitro* tissue models is essential for their successful validation and routine use in regulatory assays and research programs across the cosmetics and chemical industries. These requirements have led to an almost exclusive dependence on commercially produced models for regulatory and industrial applications. However, two long-standing limitations of commercial models persist: the proprietary nature of production protocols and media formulations, and the requirement to ship tissues from the manufacturing facility to the end-user laboratory.

Proprietary “black box” methods hinder experimental design, complicate data interpretation and troubleshooting, and restrict opportunities for innovation and improvement. Shipping introduces an additional layer of variability and risk. Rough handling, seasonal temperature extremes, fluctuations in temperature, pressure and radiation exposure during air transport, weather-related delays, and potential x-ray exposure, mishandling or delays during Customs inspections can compromise tissue integrity, making international shipments particularly challenging or even unfeasible.

The work presented here addresses these barriers by providing detailed media compositions and production protocols. While some of the medium components that were obtained from commercial sources, such as the FGM-2 (Lonza), as well as the Supplement S7 and the Medium 154 and EpiLife basal media (ThermoFisher) are proprietary, as much detail as possible has been provided for the DDM, ESM and EDM formulations. These will enable companies and research laboratories to establish in-house production of fully animal-free FT-Skin models, reducing reliance on proprietary commercial skin models. This approach increases flexibility in experimentation, supports innovation and improvements, and eliminates the logistical and quality issues associated with shipping pre-manufactured tissues.

### 4.3 Mechanistic insights

Several noteworthy mechanistic insights and observations related to development of both the dermal and epidermal components of the FT-Skin tissues were provided by the currently reported results.

#### 4.3.1 Control of fibroblast proliferation and ECM deposition in the dermal component

ECM proteins including fibronectin, laminins and other components secreted by both dermal fibroblasts and epidermal keratinocytes interact with basal keratinocytes at the dermal-epidermal junction via integrin binding (Hinz, 2015). These interactions mediate adhesion, spreading, maintenance of stem cell fate, stratification and differentiation of keratinocytes (Watt and Fujiwara, 2011). Binding of laminin 332 to integrins localized in keratinocyte hemidesmosomes initiates assembly of the basement membrane structures that provide the mechanical framework for adhesion of the epidermis to the dermis. A polymerized network of integrin-bound laminin forms the backbone of the basement membrane, and recruits a secondary network of collagen IV, nidogen and other components, which cross-link and stabilize the lamina densa structure (Matlin et al., 2017).

A comparison of the dermal constructs produced by the 14-day vs. 3-day dermal culture protocols shows that the level of fibroblast proliferation and ECM production is largely determined by the duration of the initial dermal development period. Following removal of DDM and subsequent transition to ESM and EDM, the fibroblast levels remain steady during the remainder of the culture period, with only slight additional increases in proliferation and ECM deposition during the remaining ALI culture. It is also noteworthy that the thick fibroblast layer produced by the 14-day protocol induced a pronounced vertical, but not horizontal compression of the electrospun scaffold. Similar results were noted by Weigel et al. (2022) using electrospun polyamide six and PCL electrospun scaffolds.

These results indicate that the 3-day dermal development protocol may provide the most relevant FT-Skin model to reproduce the normal physiological state and homeostasis of *in vivo* human skin (i.e., stable epidermal thickness and quiescent dermal fibroblasts). However, the FT-Skin model protocol is also adaptable to accommodate the goals of experiments related to skin aging and wound healing, for example, by altering the number of fibroblasts initially seeded, the duration of the dermal culture period, or the age and passage number of the donor fibroblasts.

#### 4.3.2 Effect of epidermal medium formulations in directing differentiation and lifespan of the epidermal component

The current work highlights the effect of epidermal media formulations in influencing the morphological differentiation and long-term stability of FT-Skin models. A longstanding problem experienced by many currently available *in vitro* skin tissue models is a short lifespan. This limited lifespan is believed to be due to cellular senescence and/or dysregulated differentiation accompanied by loss of proliferative potential of the basal keratinocytes (Dos Santos et al., 2015). In this scenario, following a brief period of apparent normal differentiation and stable homeostasis, the thickness of the epidermal layer rapidly deteriorates until the viable basal layer ultimately disappears altogether, leaving little more than non-viable stratum corneum layers (Tham, et al., 2025). Examples of this phenomenon were shown from experiments conducted with an EDM formulation based on a DMEM/KGM® Gold keratinocyte growth medium. Similar results from the use of a KGM® Gold-based skin differentiation medium were reported by Lange et al. (2016). Thus, commercially available keratinocyte growth media that have been optimized to promote cell proliferation are often not ideal for skin model differentiation. EpiLife and Medium 154 media supplemented with HKGS appear to be uniquely compatible with both keratinocyte proliferation as well as skin model differentiation. Because the listed components of the basal media and supplements of the various media formulations are very similar, the key factors responsible for driving the epithelial lifespan are likely due to differences in the concentrations of basal media components or supplements.

### 4.4 Limitations and future directions

The current work demonstrates the feasibility of implementing FT-Skin model production on electrospun scaffolds using fully animal-free culture media. Several limitations remain to be addressed, including a need for more complete molecular characterization of the FT-Skin model, further understanding of the mechanisms governing epidermal barrier formation and tissue lifespan, and validation of the FT-Skin model for specific assays and applications. Development of fully defined media formulations that eliminate reliance on HPL, HSA and proprietary supplements such as Supplement S7 also remains a key future objective.

This study contributes to a growing foundation for further advancement of animal-component-free FT-Skin models. Future efforts will focus on further extension of tissue lifespan, performing deeper quantitative cellular and molecular analyses, and validating model performance in regulatory assays such as skin irritation, corrosion, genotoxicity, and sensitization testing. Beyond regulatory applications, these FT-Skin models offer substantial potential for research in skin aging, wound healing, and diseases involving dermal–epidermal interactions, including psoriasis and atopic dermatitis. In addition, the stability and enhanced lifespan of *in vitro* FT-Skin models based on electrospun scaffolds should facilitate development of next-generation models that incorporate additional capabilities and physiological features such as hypodermis, innervation, immune components, and vascularization. Finally, the electrospun scaffolds and core methodologies for generating animal-free subepithelial stromal constructs described here should be broadly adaptable for engineering other animal-free full-thickness *in vitro* epithelial tissue models, including those of the ocular surface, airway, intestine and others.

## Data Availability

The raw data supporting the conclusions of this article will be made available by the authors, without undue reservation.

## Footnotes

1 Use of Triton X-100 has been banned for most industrial and large-scale, commercial, and research applications in the EU. It was placed on the REACH Authorization List (Annex XIV) due to its biodegradation into toxic, endocrine-disrupting chemicals, with a sunset date of 4 January 2021, restricting its use without special authorization. Nevertheless, as of the current date, Triton X-100 is still in use for ET50 testing in the EU following OECD TG 439 and other OECD TGs. TX-100 alternates have been suggested but have not been evaluated or adopted for use in OECD skin model ET50 testing.
