# Supplementary material for: Next-generation full-thickness human skin models produced using 3D electrospun scaffolds and animal-component-free culture media
Source: Front Toxicol. 2026 May 13;8:1639389. doi: 10.3389/ftox.2026.1639389 (PMC13211859; doi:10.3389/ftox.2026.1639389)
Supplement: Supplementary file 1 [file DataSheet1.pdf]

Supplementary Material

1 Supplementary Information

1.1 Initial medium optimization for production of FT-Skin tissues on electrospun scaffold inserts

Preliminary experiments had found that dermal constructs produced by culturing NHDF within Bio-Spun™ PET scaffolds using fibroblast growth medium containing 2 % FBS (FGM-2, Lonza) supplemented with 50 µg/ml of 2-phospho-L-ascorbic acid did not produce adequate extracellular matrix to support development of a well-organized epithelium (not shown). Thereafter, matrix promoting growth factors including transforming growth factor β1 (TGF-β1) (Hinz, 2015) and connective tissue growth factor (CTGF) (Chen at al., 2020) were evaluated for their ability to improve development of the dermal component and subsequent epidermal development. These growth factors are known to stimulate fibroblast production of ECM components including fibronectin, laminins and other components (Hinz, 2015), which mediate adhesion and spreading, maintenance of stem cell fate, stratification and differentiation of keratinocytes via integrin binding (Watt & Fujiwara, 2011).

Two basal EDM formulations were also concurrently evaluated. The first consisted of complete Medium 154 supplemented with Ca^{2+} to a final concentration of 1.2 mM, an approach commonly utilized to produce differentiated *in vitro* skin models (Rosdy and Clauss (1990). The second consisted of Medium 154 blended 1:1 with DMEM, an approach similar to that used by Gray et al. (1996), wherein a 1:1 blend of airway growth medium with DMEM is used to produce differentiated airway epithelial models. The M154:DMEM blend was supplemented with HKGS at the same concentration as the unblended complete M154 medium (i.e. 1:100). In order to promote robust barrier properties, both basal formulations were further supplemented with 2-phospho-L-ascorbic acid, isoproterenol, enhanced levels of amino acids required for phospholipid, sphingolipid synthesis and lipid metabolism (L-serine and L-carnitine), fatty acid-free BSA, and stratum corneum precursor lipids (Boyce and Williams,1993; Ponec et al. 1997). The media were also supplemented with ilomastat, a matrix metalloproteinase inhibitor, in order to promote development of a stable basement membrane at the dermal-epidermal junction (Amano et al., 2001; Iriyama et al., 2018, 2019; Varkey et al., 2014). A schematic summary of the medium conditions tested is shown in Supplemental Figure 1.

Evaluation of H&E-stained tissue constructs confirmed that FGM-2 medium supplemented with 2-phospho-L-ascorbic acid alone did not support optimum development of a full-thickness skin tissue. The dermis was robust in the sense that it prevented the keratinocytes from infiltrating into the scaffold, yet the keratinocytes did not spread and fully cover the dermal component, and the epithelial layers were poorly organized. Further addition of CTGF alone also failed to support development of a complete and uniform epithelial component. However, dermal constructs initiated in medium containing TGF-β1, whether alone or in combination with CTGF, produced a uniform, well-developed and well-organized epithelium (Supplemental Figure 2A). There was no discernable difference in the effects of the 5 or 10 ng/mL levels of TGF-β1 (not shown). The higher level was adopted for ongoing use to ensure that an adequate effect was reproducibly achieved.

Regardless of the presence of TGF-β1 in the dermal development medium (DDM), tissue constructs produced using the unblended M154-based epidermal differentiation medium (EDM) produced tissues with poor coverage, organization and morphology compared to the M154:DMEM EDM blend (Supplemental Figure 2B).

EDM prepared with blends containing other commercially available brands of keratinocyte growth media and supplements produced FT-Skin tissues that underwent rapid depletion of the cuboid basal keratinocytes and deterioration of the epithelial component (Supplemental Figure 3).

1.2 Evaluation of FT-Skin tissue development with multiple fibroblast/keratinocyte donor pairs

Additional donor pairs (Supplemental Table 1) were evaluated using the general FT-Skin model production protocol (main manuscript section 2.7.1, Figure 3) with 150 µm thick PET scaffolds in a 24-well HTS format (Figure 2B). Following scaffold activation, NHDF were seeded in a volume of 100 µL onto the apical surface of the scaffolds in DDM (containing FBS) at a density of 300,000 cells/cm^2. This seeding density produced a robust dermis within 4 days for all of the NHDF donors.

On Day 4 following NHDF seeding, NHEK donor lots were harvested (~80 % confluence) for seeding onto the fibroblast-populated scaffolds. The DDM was replaced with Epidermal Submerged Medium (ESM), consisting of Medium 154 supplemented with 50 µg/ml of 2-phospho-L-ascorbic acid (1.0 mL in the well underneath each scaffold). HEKn or NHEK were than seeded onto the apical surfaces of the fibroblast-populated scaffolds (200K cells in 200 µL of HKGS-containing ESM per insert). The following day, the culture medium was replaced with fresh ESM (2.5 mL/insert) to completely submerge the cultures.

Tissues were airlifted after 3 days of submerged culture by using the 24-well HTS ALI lifter (Figure 2B). The ALI lifter was inserted between the scaffold plate and the 24-well reservoir plate. A fresh 24-well reservoir plate was used at the time of airlift to avoid overgrowth of any fibroblasts or keratinocytes in the reservoir that may have accumulated during the previous seeding/culturing steps (this was generally not a problem, but was randomly observed in isolated wells from time to time). The ESM was replaced with M154:DMEM-based EDM (HKGS-containing). The 24-well HTS ALI lifter allows the cultures to be fed with 3.0 mL/well of EDM beneath each tissue. The apical surfaces of the tissues were left dry from this point onward. The cultures were fed every other day, with FT-Skin tissue samples periodically removed every 4 days, beginning at day 8 after ALI culture, and fixed for histological assessment (Supplemental Figure 7).

Supplementary Figures and Tables

Supplemental Figure 1. Optimization of dermal and epidermal media formulations.

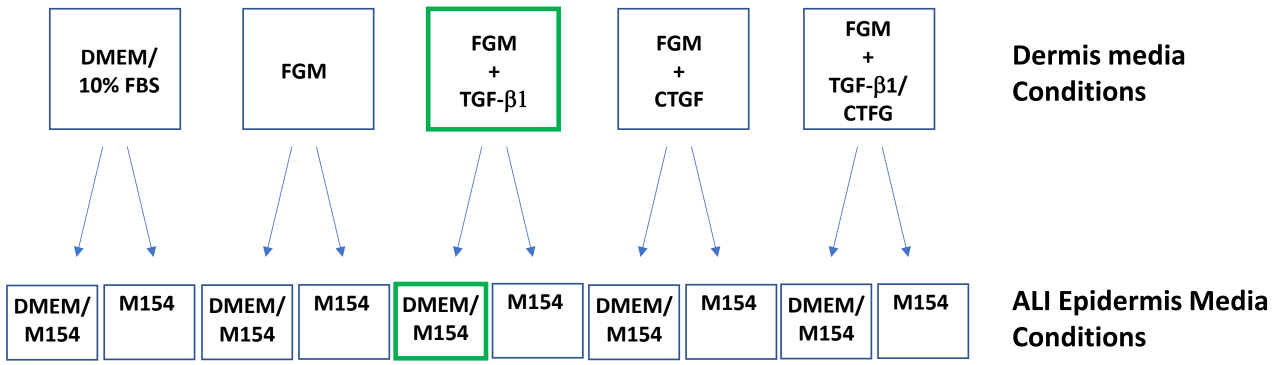

Dermal and epidermal medium formulations were tested concurrently to identify the optimal media formulations for FT-Skin model development. DDM formulations included: DMEM supplemented with 10% FBS; FGM-2; FGM-2 supplemented with TGF-β1 (5 and 10 ng/mL); FGM-2 supplemented with CTGF (10 and 20 ng/mL); FGM-2 supplemented with TGF-β1 and CTGF. All of the FGM-2 formulations contained 2 % FBS. All of the dermal media formulations were also supplemented with 2-phospho-L-ascorbic acid (50 µg/mL). ESM consisting of Medium 154 supplemented with HKGS (1/100 dilution) was utilized for all cultures during the epidermal submerged culture phase. EDM formulations utilized during the ALI culture phase included: Medium 154 supplemented with HKGS (1/100 dilution); a blend of DMEM (high glucose) and Medium 154 (50:50) also supplemented with HGKS (1/100 dilution). All of the epidermal differentiation media formulations were also supplemented with 2-phospho-L-ascorbic acid (50 µg/mL). All of the epidermal submerged and differentiation media formulations were also supplemented with 2-phospho-L-ascorbic acid (50 µg/mL). The optimal media combination determined as described in Supplemental Section 1.1 above is indicated by the green boxes.

Supplemental Figure 2. Histologic evaluation of FT-Skin tissues produced with various medium conditions.

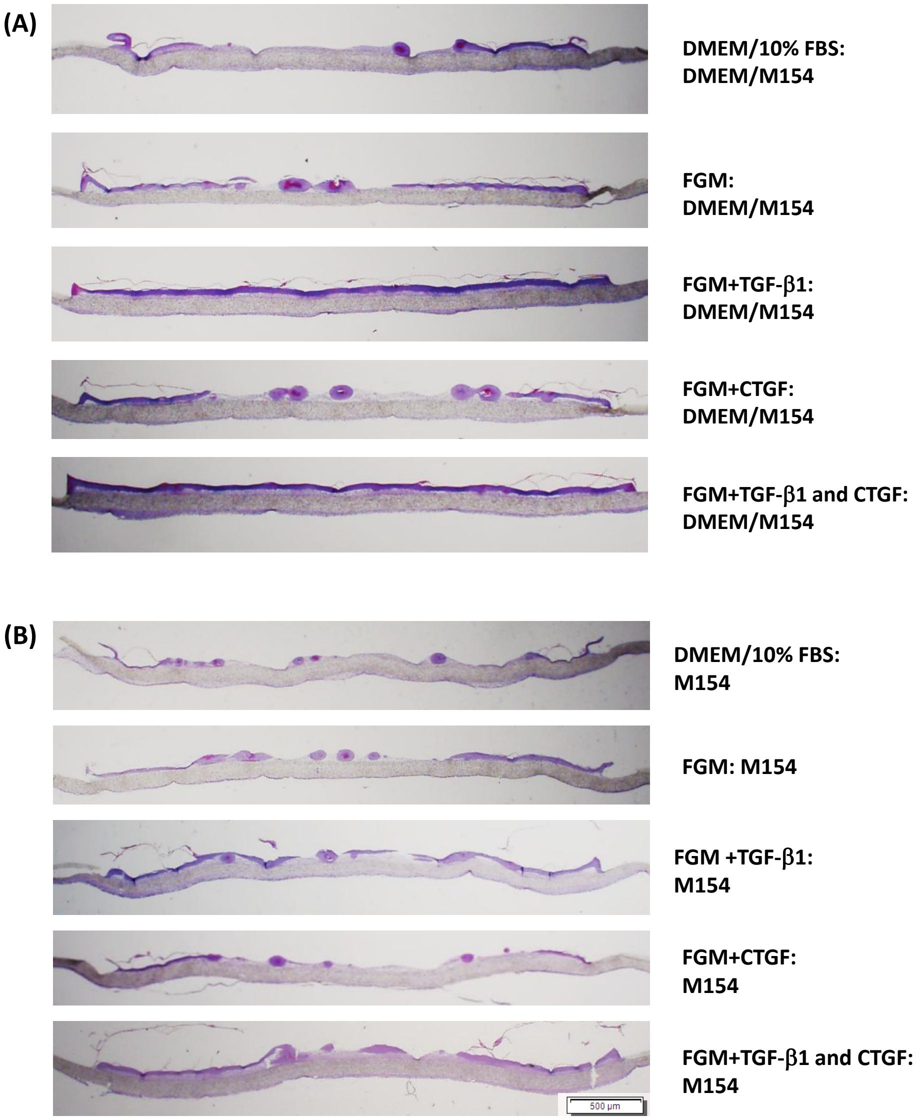

A: H&E-Stained cross sections of FT-Skin tissues produced with the indicated dermal media and growth factors and DMEM/M154-based EDM. FGM-2 was formulated with FBS. NHDF were seeded at a density of 150,000 cells/cm^2, and subsequently cultured for a period of 2 weeks prior to seeding of NHEK. Medium 154-based ESM, EDM were formulated with HKGS. TGF-β1 concentration was 10 ng/mL. CTGC concentration was 20 ng/mL. H&E-stained cross section is shown at day 14 after ALI culture. **B:** H&E-Stained cross sections of FT-Skin tissues produced with the indicated dermal media and growth factors and unblended M154-based EDM. For both A and B conditions, the dermis was robust in the sense that it prevented the keratinocytes from infiltrating into the scaffold. However, the keratinocytes did not spread and fully cover the dermal component, and the epithelial layers were incomplete and poorly organized. Uniform epidermal development and wall-to wall coverage of the epithelial layer were only obtained with dermal medium supplemented with TGF-β1 followed by DMEM/M154 based EDM. Scale bar = 500 µm.

Supplemental Figure 3. Histologic evaluation of FT-Skin tissues produced with ESM and EDM formulated with alternate KGM brand.

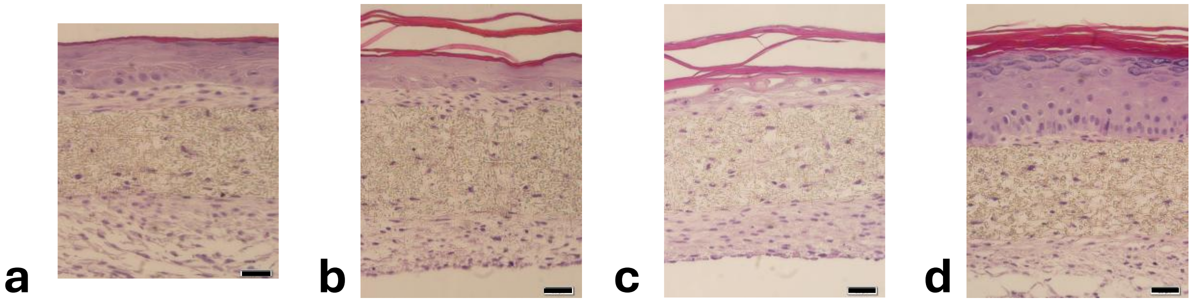

D8ALI **D12ALI** **16ALI** **D16ALI**

ESM/EDM formulated with alternate commercial KGM Brand

M154-based ESM/EDM

H&E-stained cross sections of FT-Skin tissues produced using ESM and EDM formulated with an alternate brand of commercially available KGM (General FT-Skin model development protocol with 14-day dermal culture). The cultures display rapid depletion of cuboid basal keratinocytes and deterioration of the epithelial component within 16 days after initiation of ALI culture. A comparative control sample produced with DMEM/M154-based ESM and EDM is shown at Day 16 ALI (D16ALI). Scale bar = 20 μm.

Supplemental Figure 4. Van Gieson’s stain for collagen deposition in long-term (14-day) *vs.* short-term (3-day) dermal culture.

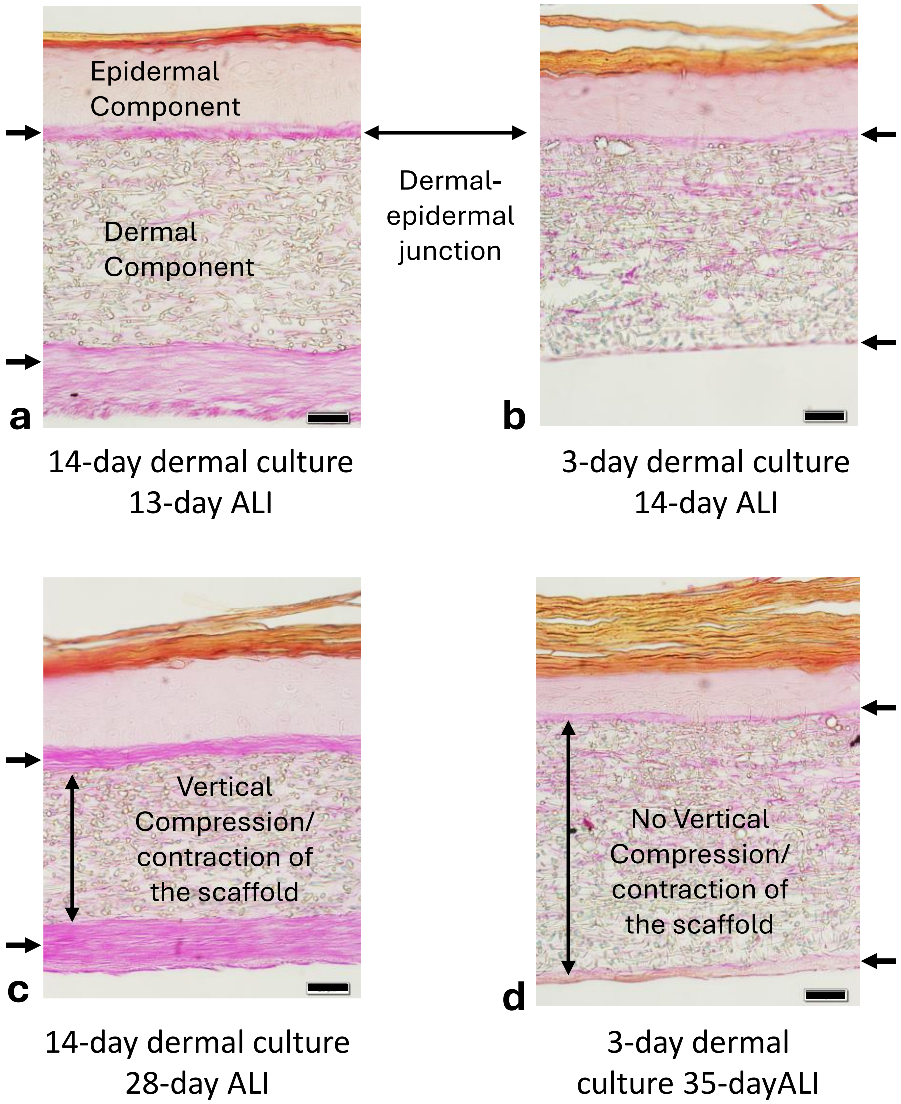

Collagen deposition in FT-Skin tissues produced by the General FT-Skin model production protocol (main manuscript Section 2.7.1) with 14-day dermal culture (Panels a,c) and 3-week FT-Skin model production protocol (main manuscript Section 2.7.2) with 3-day dermal culture (Panels b,d) was assessed by Van Gieson’s Stain after 13-14 days (Panels a,b) and 28-35 days (Panels c,d) days of ALI culture. Collagen deposition is seen within the electrospun scaffold as well as at the top surface of the scaffold at the dermal-epidermal junction and the lower surface of the scaffold. Long-term dermal development produced a thicker deposition at the upper and lower scaffold surfaces (arrowhead), and caused compression/contraction of the scaffold in the vertical, but not horizontal direction. The thickness of collagen layer deposition at the upper and lower scaffold surfaces was determined primarily by the time of dermal development (panels a,c vs. c,d). The short-term protocol was found to produce a robust dermal component that supported epithelial development without keratinocyte infiltration into the scaffold within 3 days (Panels b,d). Scale bar = 20 µm.

Supplemental Figure 5. Keratinocyte infiltration into electrospun scaffold due to inadequate extracellular matrix deposition.

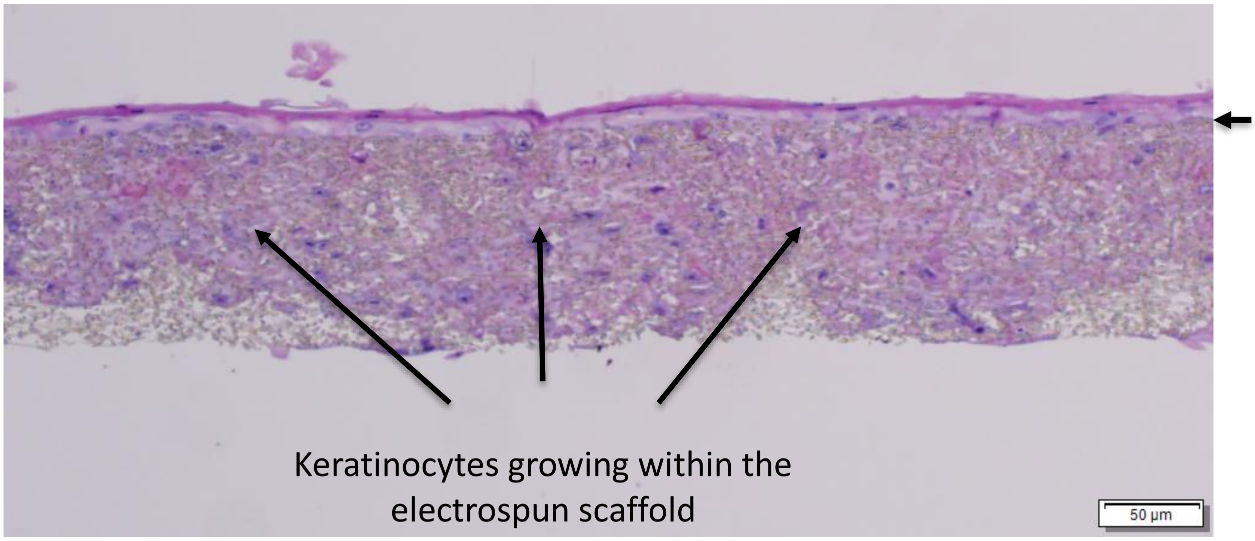

H&E cross section of skin model without adequate dermal development. NHDF (150K/cm^2) were cultured using the General FT-Skin model protocol (main manuscript Section 2.7.1) for only 3 days prior to seeding NHEK. The low seeding density of NHDF did not produce adequate ECM within 3 days. Keratinocytes migrated into the scaffold rather than forming a stratified epithelial tissue above the scaffold. The top surface of the scaffold is shown by the arrowhead.

Supplemental Figure 6. Histologic evaluation of FT-Skin tissues produced from PDLGA/PLLA bilayer scaffolds.

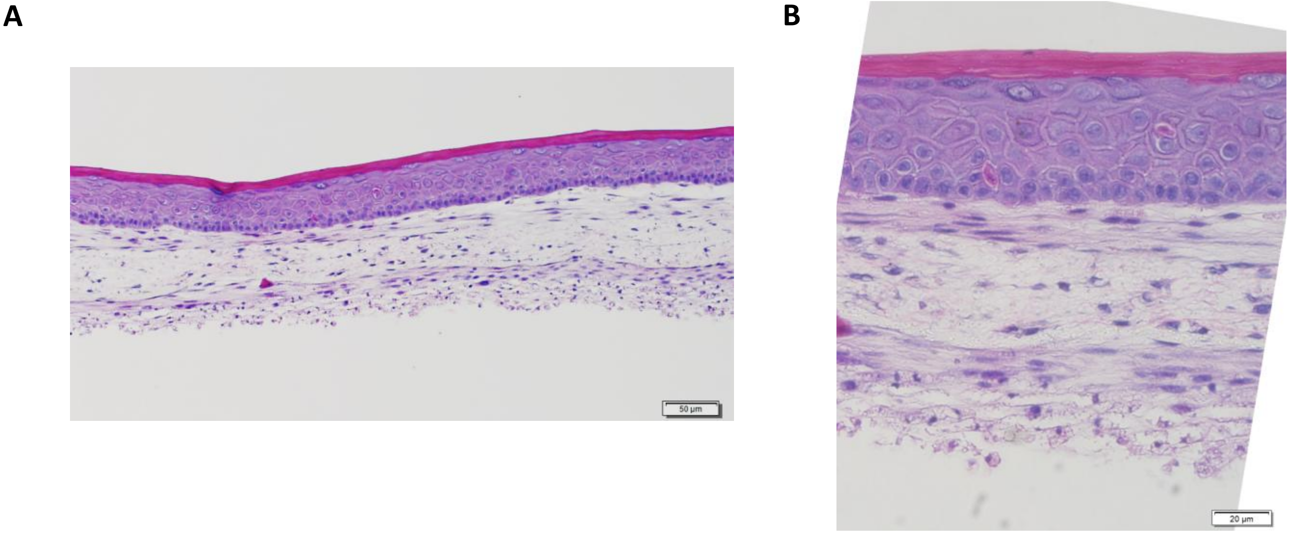

Biodegradable PDLGA/PLLA bilayer scaffolds were evaluated for ability to support FT-Skin model development using the General FT-Skin model protocol (main manuscript Section 2.7.1). DDM was formulated with FBS. NHDF were seeded at a density of 150,000 cells/cm^2, and subsequently cultured for a period of 2 weeks prior to seeding of NHEK. Medium 154-based ESM, EDM were formulated with HKGS. H&E-stained cross section is shown at day 14 after ALI culture. Panel A scale bar = 50 µm. Panel B scale bare = 20 µm.

Supplemental Table 1.

Supplemental Table 1. Donor Information for NHDF and HEKn/NHEK

Cell Type	Donor ID#	Source	Donor Demographics	Final Passage Used
NHDF	19TL149597	Lonza	Neonatal, Male	P6
NHDF	*22TL261645	Lonza	Neonatal, Male	P5
NHDF	**22TL346464	Lonza	Neonatal, Male	P5
NHDF	22TL346467	Lonza	38 yr old, Black, Adult Female	P4
HEKn	2491219	ThermoFisher	Neonatal, Male, Light	P4
HEKn	2825951	ThermoFisher	Neonatal, Male, Light	P4
NHEK	**22TL346482	Lonza	Neonatal, Male	P4
NHEK	*22TL261644	Lonza	Neonatal, Male	P4

To verify the robustness of the FT-Skin model production protocol and media formulations, testing was conducted with multiple fibroblast and keratinocyte donors. Sources, donor demographics and the final passage of cells used for FT-Skin model production are given above. *,**NHDF/NHEK are from the same donor.

Supplemental Figure 7. Evaluation of FT-Skin production protocol and medium formulations with multiple NHDF and HEKn/NHEK donor combinations.

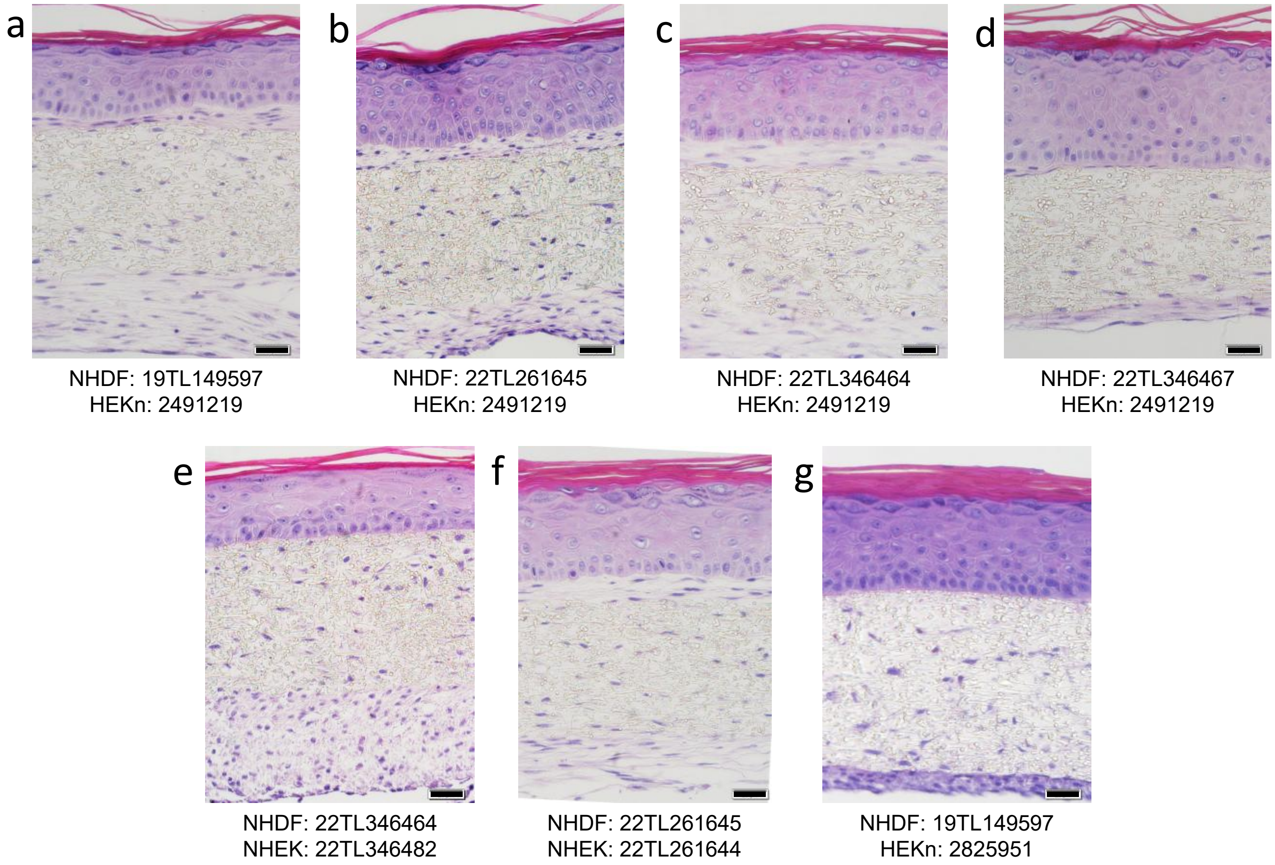

To verify the robustness of the protocols and media formulations, testing was conducted with additional fibroblast/keratinocyte pairs. Four NHDF and 4 HEKn/NHEK donors were tested in various pairings using the General FT-Skin model production protocol (main manuscript Section 2.7.1, Figure 3). NHDF were seeded at a density of 300,000 cells/cm^2, and subsequently cultured for a period of 4 days prior to seeding of NHEK at a density of 606,000 cells/cm^2. Complete donor information is given in Supplemental Table 1. H&E-stained cross sections show that comparable morphology, epidermal thickness and stratum corneum development is evident among all donor pairs at 14 days of ALI culture. Scale bar = 20 µm

Supplemental Table 2. Quantitative Evaluation of Epidermal Thickness Over Time.

	Epidermal Thickness (Microns +/- Standard Deviation)				
Day*	3W3D/HPL:M154/S7[a]	3W3D/HPL:EpiLife/S7[b]	3W3D/HPL:M154/HKGS[c]	G14D/FBS:M154/HKGS[d]	G14D/FBS:EpiLife/HKGS[e]
12	30.44 +/- 2.23	48.30 +/- 3.23	43.74 +/- 1.36		
13-14	31.69 +/- 1.38	45.55 +/- 3.00	47.52 +/- 1.69	41.44 +/- 1.50	47.33 +/- 3.36
16-18	34.14 +/- 4.14	44.99 +/- 2.87	38.34 +/- 1.36	47.25 +/- 4.50	49.86 +/- 2.86
21-23	37.57 +/- 1.08	50.35 +/- 1.87	44.46 +/- 2.60	44.85 +/- 4.00	44.02 +/- 3.58
26	26.93 +/- 1.88		31.28 +/- 1.21		
28-30	18.10 +/- 1.22	47.73 +/- 4.02	34.83 +/- 1.88	45.65 +/- 3.50	41.52 +/- 3.27
36			31.10 +/- 1.05	34.35 +/- 2.90	

A set of 5 independent experiments assessed how dermal culture duration as well as ESM and EDM media formulations affected morphological integrity and epidermal thickness over 36 days of ALI culture. The thickness of epidermal layers was measured at 4 locations within a representative field of view. Thickness values shown are averages +/- standard deviation for each sample. **3W3D/HPL:M154/S7** = 3-week protocol with 3-day dermal culture using HPL-based DDM and Medium 154/Supplement S7-based ESM and EDM. **3W3D/HPL:EpiLife/S7** = 3-week protocol with 3-day dermal culture using HPL-based DDM and EpiLife/Supplement S7-based ESM and EDM. **3W3D/HPL:M154/HKGS** = 3-Week protocol with 3-day dermal culture using HPL-based DDM and Medium 154/HKGS-based ESM and EDM. **G14D/FBS:M154/HKGS** = General protocol with 14-day dermal culture using FBS-based DDM and Medium 154/HKGS-based ESM and EDM. **G14D/FBS:EpiLife/HKGS** = General protocol with 14-day dermal culture using FBS-based DDM and EpiLife/HKGS-based ESM and EDM.

References

Amano, S., Akutsu, N., Matsunaga, Y., Nishiyama, T., Champliaud, M. F., Burgeson, et al. (2001). Importance of balance between extracellular matrix synthesis and degradation in basement membrane formation. *Experimental cell research*, *271*(2), 249–262. https://doi.org/10.1006/excr.2001.5387

Boyce, S. T., & Williams, M. L. (1993). Lipid supplemented medium induces lamellar bodies and precursors of barrier lipids in cultured analogues of human skin. *The Journal of investigative dermatology*, *101*(2), 180–184. https://doi.org/10.1111/1523-1747.ep12363678

Chen, Z., Zhang, N., Chu, H. Y., Yu, Y., Zhang, Z. K., Zhang, G., et al. (2020). Connective Tissue Growth Factor: From Molecular Understandings to Drug Discovery. *Frontiers in cell and developmental biology*, *8*, 593269. https://doi.org/10.3389/fcell.2020.593269

Gray, T. E., Guzman, K., Davis, C. W., Abdullah, L. H., & Nettesheim, P. (1996). Mucociliary differentiation of serially passaged normal human tracheobronchial epithelial cells. *American journal of respiratory cell and molecular biology*, *14*(1), 104–112. https://doi.org/10.1165/ajrcmb.14.1.8534481

Hinz B. (2015). The extracellular matrix and transforming growth factor-β1: Tale of a strained relationship. *Matrix biology: journal of the International Society for Matrix Biology*, *47*, 54–65. https://doi.org/10.1016/j.matbio.2015.05.006

Iriyama, S., Matsuura-Hachiya, Y., & Tsunenaga, M. (2018). Influence of epidermal basement membrane integrity on cutaneous permeability barrier function. *Journal of dermatological science*, *91*(1), 110–112. https://doi.org/10.1016/j.jdermsci.2018.04.004

Ponec, M., Weerheim, A., Kempenaar, J., Mulder, A., Gooris, G. S., Bouwstra, J., et al. (1997). The formation of competent barrier lipids in reconstructed human epidermis requires the presence of vitamin C. *The Journal of investigative dermatology*, *109*(3), 348–355. https://doi.org/10.1111/1523-1747.ep12336024

Rosdy, M., & Clauss, L. C. (1990). Terminal epidermal differentiation of human keratinocytes grown in chemically defined medium on inert filter substrates at the air-liquid interface. *The Journal of investigative dermatology*, *95*(4), 409–414. https://doi.org/10.1111/1523-1747.ep12555510

Varkey, M., Ding, J., & Tredget, E. E. (2014). Superficial dermal fibroblasts enhance basement membrane and epidermal barrier formation in tissue-engineered skin: implications for treatment of skin basement membrane disorders. *Tissue engineering. Part A*, *20*(3-4), 540–552. https://doi.org/10.1089/ten.TEA.2013.0160

Watt, F. M., & Fujiwara, H. (2011). Cell-extracellular matrix interactions in normal and diseased skin. *Cold Spring Harbor perspectives in biology*, *3*(4), a005124. https://doi.org/10.1101/cshperspect.a005124
